# Cardiovascular mortality risk in patients with ovarian cancer: a population-based study

**DOI:** 10.1186/s13048-024-01413-4

**Published:** 2024-04-25

**Authors:** Ze-Lin Hu, Ying-Xue Yuan, Meng-Yi Xia, Ying Li, Ying Yang, Sheng-Nan Wang, Xuan-Zhu Meng, Mo-Ying Sun, Ning Wang

**Affiliations:** The Second Hospital of Dalian Medical University, Dalian, China

**Keywords:** Ovarian cancer, Cardiovascular mortality, SEER database, Overall survival

## Abstract

**Objectives:**

Ovarian cancer (OC) can occur at different ages and is affected by a variety of factors. In order to evaluate the risk of cardiovascular mortality in patients with ovarian cancer, we included influencing factors including age, histological type, surgical method, chemotherapy, whether distant metastasis, race and developed a nomogram to evaluate the ability to predict occurrence. At present, we have not found any correlation studies on cardiovascular death events in patients with ovarian cancer. This study was designed to provide targeted measures for effective prevention of cardiovascular death in patients with ovarian cancer.

**Methods:**

Kaplan–Meier analysis and multivariable Cox proportional model were performed to evaluate the effectiveness of cardiovascular diseases on overall survival (OS) and ovarian cancer‐specific survival (OCSS). We compared multiple groups including clinical, demographic, therapeutic characteristics and histological types. Cox risk regression analysis, Kaplan–Meier survival curves, and propensity score matching were employed for analyzing the data.

**Results:**

A total of 88,653 ovarian cancer patients were collected, of which 2,282 (2.57%) patients died due to cardiovascular-related diseases. Age, chemotherapy and whether satisfactory cytoreduction surgery is still the most important factors affecting the prognosis of ovarian cancer patients, while different histological types, diagnosis time, and race also have a certain impact on the prognosis. The newly developed nomogram model showed excellent predictive performance, with a C-index of 0.759 (95%CI: 0.757–0.761) for the group. Elderly patients with ovarian cancer are still a high-risk group for cardiovascular death [HR: 21.07 (95%CI: 5.21–85.30), *p* < 0.001]. The calibration curve showed good agreement from predicted survival probabilities to actual observations.

**Conclusion:**

This study found that age, histology, surgery, race, chemotherapy, and tumor metastasis are independent prognostic factors for cardiovascular death in patients with ovarian cancer. The nomogram-based model can accurately predict the OS of ovarian cancer patients. It is expected to inform clinical decision-making and help develop targeted treatment strategies for this population.

**Supplementary Information:**

The online version contains supplementary material available at10.1186/s13048-024-01413-4

## Highlights

• When patients with ovarian cancer are diagnosed, most are at an advanced stage and are diagnosed after menopause.

• The nomogram is a powerful prediction model for the occurrence of CVM in elderly patients with ovarian cancer.

• The model has good predictive ability and provides personalized treatment for the prevention of CVM in elderly OC patients.

**Supplementary Information:**

The online version contains supplementary material available at10.1186/s13048-024-01413-4

## Introduction

In 2020, the global estimated new cases of ovarian cancer (OC) was 313,959; (the death toll reached 207,252); the death rate was 66.01% [1]. OC is diagnosed at an advanced stage, with high recurrence and mortality rates owing to the lack of cancer-specific symptoms and effective screening tools [2]. Meanwhile, with the continuous economic and social development, the number of deaths caused by cardiovascular diseases is gradually increasing worldwide. Studies have been conducted to show that cardiovascular mortality (CVM) increased by 2.1% over a 10-year period from 2007 [3].

It is well known that the risk of developing CVM varies dramatically between malignancies. Patients with colorectal cancer and endometrial cancer have 11.7- and 8.8-fold higher risk of CVM than the general population, respectively [4, 5].The incidence of CVM in prostate cancer and gastroenteropancreatic neuroendocrine neoplasms is 2.05 and 0.92 times higher in the first month and 7–12 months after diagnosis, respectively, than in the general population [6, 7]. Several published studies have shown that the risk of CVM in cancer patients is significantly higher than that of the rest of the general population [5, 8].

To our knowledge, no predictive prediction of cardiovascular risk in patients with ovarian cancer has been retrieved. In this study, we described the prediction and analysis of causes of death from multiple perspectives and identified independent risk factors for cardiovascular death in patients with ovarian cancer, which has guiding significance for us to adopt individualized treatment plans for specific groups, and establish individualized surveillance strategies and interventions.

## Materials and Methods

### Data and patients selection

Information on all ovarian cancer patients is available from the Surveillance, Epidemiology, and End Results (SEER) database (usig) SEER*Stat software (National Cancer Institute, Bethesda, MD, USA, version 8.4.1.2, Database: Incidence-SEER Research Plus Data, 18 Registries (excl AK), Nov 2020 Sub (2000–2018)). Our publicly available data from the SEER database do not require ethics committee approval.

On this basis, we identified a total of 88,653 ovarian cancer patients, and the inclusion criteria were as follows: (1) Patients were diagnosed clinically and/or histologically, (2) Variables: age, race, year of diagnosis, summary stage, survival months, chemotherapy recode, surgery, histological type. The exclusion criteria were: (1) Identified by autopsy or death certificate, (2) Unknown race, age, (3) Unknown summary stage, (4) Unknown chemotherapy, (5) No positive histology, (6) Unknown cause of death, (7) Unknown surgery, (8) Unknow type of reporting source.

Death from cardiovascular disease is the primary endpoint of our study. CVD is defined by the SEER database and includes the following six items: (1) Diseases of the heart, (2) Hypertension without heart disease, (3) Cerebrovascular diseases, (4) Atherosclerosis, (5) Aortic aneurysm and dissection, (6) Other diseases of the arteries, arterioles, and capillaries (Fig. S1).

We chose to include patients who were pathologically positively diagnosed with ovarian cancer from 2000 to 2017. Pathology was coded according to the International Classification of Diseases for Oncology, 3rd edition (ICD-O-3) and included the following: 8000, 8001–8005, 8010, 8011, 8012, 8013, 8014, 8015, 8020–8022, 8030–8034, 8041, 8043–8046, 8050–8053, 8060, 8070–8076, 8078, 8083, 8084, 8090–8095, 8097, 8140, 8240, 8245, 8246, 8255, 8260–8263, 8255, 8260–8263, 8310, 8313, 8320–8323, 8330–8333, 8336, 8337, 8340–8347, 8350, 8380–8384, 8440–8442, 8450, 8452, 8453, 8460–8463, 8470–8472, 8480–8482, 8490, 8500–8504, 8508, 8510, 8512–8514, 8520–8523, 8542, 8550, 8560, 8562, 8571–8575, 8590–8593, 8600–8602, 8610, 8620–8623, 8933, 8935, 8980, 8990, 9014, 9015, 9020, 9050–9052, 9071, 9080–9082, 9084, 9090, 9100–9102.

### Study variables

Detailed definitions and information about the variables are as follows: age at diagnosis (01–14, 15–29, 30–44, 45–59, 60–74, 75 + years), race (White, Black, American Indian/Alaska Native, Asian or Pacific Islander), year of diagnosis, histological type (adenocarcinoma, sarcoma, epithelial carcinoma, other types: bchondroblastic osteosarcoma, complex epithelial carcinoma, ependymoma, unclassification tumor, squamous cell carcinoma, special gonadal carcinoma, germ cell carcinoma), summary stage (unknown/unstaged, localized, regional, distant), surgery (no surgery, palliative surgery, cytoreductive surgery, other), chemotherapy recode, cause of death, and follow-up time.

### Statistical analysis

The occurrence of CVM is our primary event of concern and therefore its corresponding competing events are the cause of death due to the primary tumor, other tumors and non-other tumors. Therefore, we have adopted the following statistical approach to illustrate.

All data were obtained from the SEER (Surveillance, Epidemiology, and End Results) database with SEER*Stat software (version 8.4.1.2, National Cancer Institute, Bethesda, MD, USA), Microsoft Excel 2021 (Microsoft, Redmond, 22,082,100, USA), and R studio software (version 4.1.2) were used to complete the data analysis. Independent risk factors related to prognosis were determined by univariate Cox analysis, and a nomogram was developed based on the identified independent risk factors. The ability to discriminate between observed and predicted outcome was evaluated by Harrell's concordance index (C-index) [9]. The higher the value, the better the effect of different variables on survival outcomes. At the same time, we further used the receiver operating characteristic (ROC) curve and area under the curve (AUC) values to evaluate the prediction efficiency of the model. The usefulness of decision curve analysis for evaluating nomograms has been detailed by Vickers et al. [10]. All tests were 2-sided, and a *P*-value < 0.05 signified statistical significance.

## Results

### Patients Characteristics

**Fig. 1:**
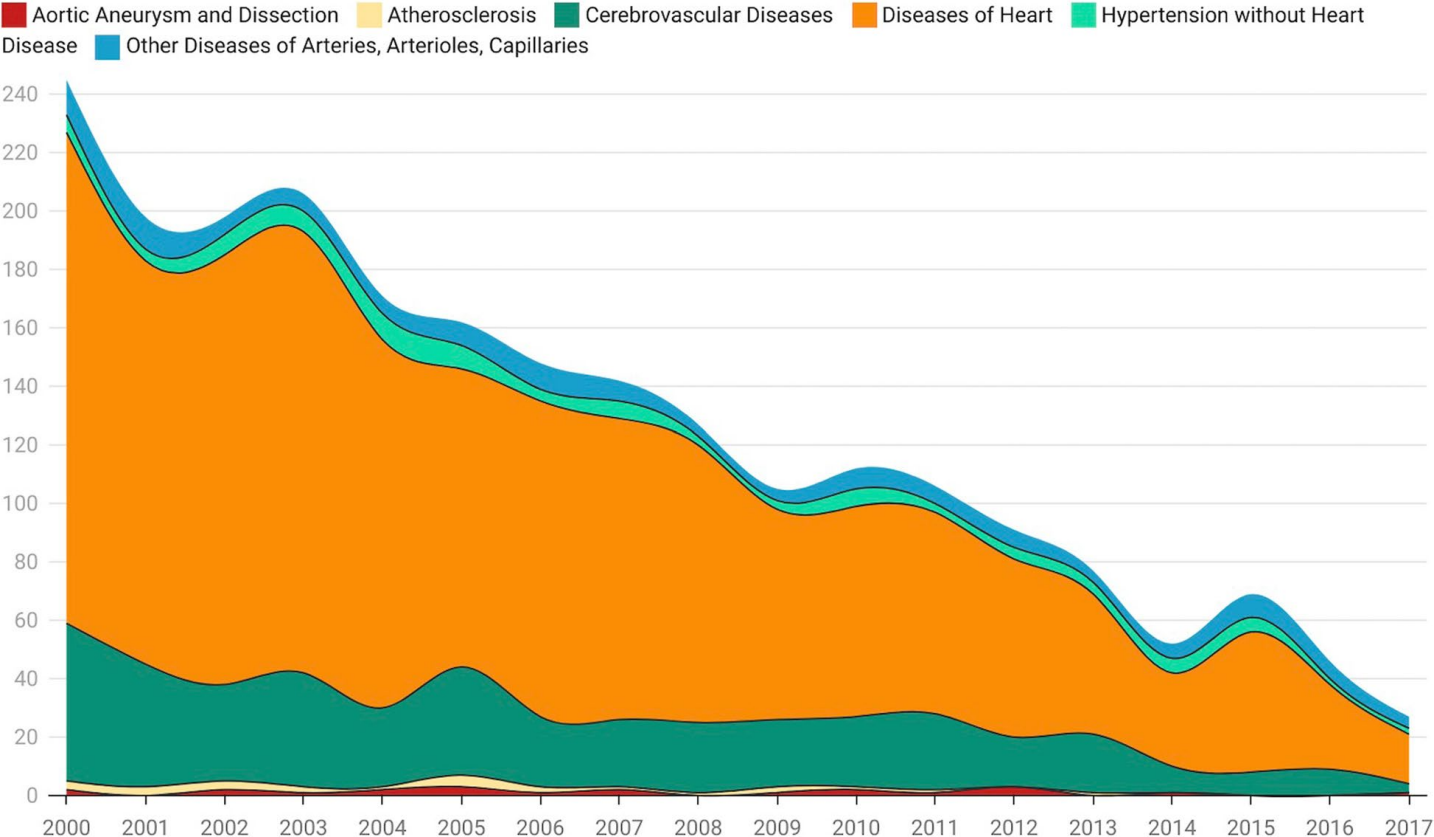
As time changes, the proportions of different CVDs indicate that disease of heart accounts for the highest proportion

**Fig. 2:**
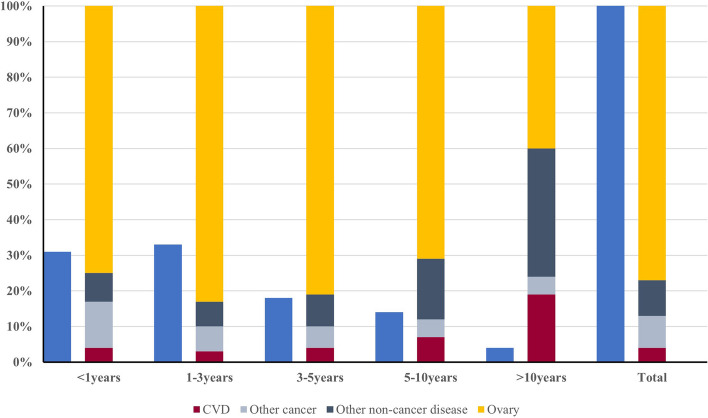
Percentage of causes of death within the specified time period after diagnosis of ovarian cancer (dark blue indicates the percentage of total deaths during each latency period)

From our data, the number of new diagnoses of ovarian cancer has not increased significantly over time (Fig. S2A). In our data, the main diagnosed age groups are 45–74 years old, including 45–59 years (32.96%) and 60–74 years (34.44%). Among the people diagnosed, the white race exceeds the vast majority (83.39%). More than half of the diagnosed people received surgical treatment (86.50%), including palliative surgery (49.12%) and cytoreductive surgery (36.39%). The histological types of ovarian cancer are composed of adenocarcinoma (84.71%), epithelial carcinoma (4.37%), sarcoma (3.65%), and other type (7.28%). Looking at chemotherapy records, more than half of ovarian cancer patients received systemic therapy (67.61%). As of the statistical time, a total of 52,139 ovarian cancer patients have died, of which 2,282 (4.38%) patients died from cardiovascular disease. At the same time, our study found that the total number of deaths from cardiovascular disease in ovarian cancer patients is declining year by year, and there are significant differences between different types of cardiovascular death (Fig. S2B). Among them, Aortic Aneurysm and Dissection (0.96%), Atherosclerosis (1.1%), Cerebrovascular Diseases (19.37%), Diseases of Heart (69.50%), Hypertension without Heart Disease (3.86%), Other Diseases of Arteries, Arterioles, Capillaries (5.21%) (Fig. S3). As time goes by, the total number of diagnosed cardiovascular deaths is declining year by year, but the proportion of ovarian cancer patients who die from heart disease is always the first among CVD (Fig. 1). Disease of heart remains the leading cause of cardiovascular death in patients with ovarian cancer. On the contrary, Aortic Aneurysm and Dissection has the smallest impact on the death of ovarian cancer patients, and can even be ignored. In addition, we calculated the proportional risk of cancer-related death. Among them, 33,394 (64.05%) ovarian cancer patients died within 3 years. The survival period of more than 10 years was significantly lower than other years. The proportion of cancer-related deaths decreased gradually at < 1 year, 1–3 years, 3–5 years, 5–10 years, and > 10 years (including OC and other cancers), while the proportion of non-cancer disease-related deaths increased gradually (including CVD and other non-cancer diseases) (Fig. 2). This is most likely due to the current lack of effective early diagnosis methods and prediction models in clinical practice, which results in the initial diagnosis of ovarian cancer at a later stage and a poor prognosis

#### Independent prognostic factors in ovarian cancer patients

In univariate Cox analysis, we found that seven variables were significantly associated with OS in patients with ovarian cancer, including age at diagnosis, race, chemotherapy, whether distant metastasis, surgical method, histological type and time of diagnosis. We fitted a normal distribution to the overall survival time of the data and found that the mean was 58.6 months (SD = 56.5) (Fig. S4). At the same time, multivariate Cox analysis also supports our judgment (Table 1). Univariate analysis showed that compared with the reference group, the death outcome was 16 times and 33 times higher respectively when the age at diagnosis was over 60 years [HR: 16.34 (95% CI: 11.35–23.51), *p* < 0.001], [HR: 33.81(95%CI: 23.49–48.68), *p* < 0.001]. If the tumor in patients with ovarian cancer does not develop distant metastasis, the survival outcome will be significantly improved [HR: 0.13 (95%CI: 0.13–0.14), *p* < 0.001]. Multivariate analysis also supports our judgment. When patients with ovarian cancer are diagnosed above the age of 75, the probability of death will be more than 10 times [HR: 11.49 (95%CI: 7.96–16.58), *p* < 0.001]. If the tumor does not metastasize distantly, the survival outcome is significantly improved [HR: 0.16 (95%CI: 0.16–0.17), *p* < 0.001]. Early detection and early treatment of ovarian cancer can significantly benefit us in improving the overall survival time of patients.

**Table 1:** Univariate and multivariate Cox proportional hazards regression analysis of the OS of ovarian cancer patients

**Characteristics**	**Univariate analysis**		**Multivariate analysis**	
	**Adjusted HR (95%CI)**	***p*-value**	**Adjusted HR (95%CI)**	***p*-value**
**Age**				
01-14 years	Reference			
15-29 years	2.69 (1.84-3.92)	<0.001	2.34 (1.61-3.42)	<0.001
30-44 years	6.72 (4.66-9.69)	<0.001	4.20 (2.91-60.07)	<0.001
45-49 years	10.81 (7.51-15.56)	<0.001	5.52 (3.82-7.96)	<0.001
60-74 years	16.34 (11.35-23.51)	<0.001	7.02 (4.87-10.13)	<0.001
75+ years	33.81 (23.49-48.68)	<0.001	11.49 (7.96-16.58)	<0.001
**Race**				
White	Reference			
Black	1.24 (1.21-1.28)	<0.001	1.25 (1.21-1.29)	<0.001
Asian or Pacific Islander	0.70 (0.68-0.73)	<0.001	0.91 (0.88-0.94)	<0.001
American Indian/Alaska Native	1.06 (0.96-1.18)	0.236	1.17 (1.05-1.30)	0.003
**Chemotherapy**				
No	Reference			
Yes	1.10 (1.08-1.12)	<0.001	0.67 (0.65-0.68)	<0.001
**Summary stage**				
Distant	Reference			
Localized	0.13 (0.13-0.14)	<0.001	0.16 (0.16-0.17)	<0.001
Regional	0.29 (0.28-0.30)	<0.001	0.36 (0.35-0.37)	<0.001
Unknown/unstaged	0.74 (0.71-0.78)	<0.001	0.47 (0.45-0.50)	<0.001
**Surgery**				
Cytoreductive surgery	Reference			
No	3.27 (3.19-3.35)	<0.001	2.71 (2.64-2.78)	<0.001
Other	1.03 (0.95-1.11)	0.525	1.28 (1.18-1.39)	<0.001
Palliative surgery	0.44 (0.43-0.45)	<0.001	0.81 (0.79-0.83)	<0.001
**ICD-O-3**	Reference			
Adenocarcinoma	Reference			
Epithelial carcinoma	1.74 (1.68-1.81)	<0.001	1.19 (1.14-1.24)	<0.001
Other types	0.41 (0.39-0.43)	<0.001	0.73 (0.69-0.76)	<0.001
Sarcoma	1.82 (1.75-1.89)	<0.001	1.64 (1.57-1.70)	<0.001
**Year of diagnose**				
2000-2005	Reference			
2006-2011	0.93 (0.91-0.95)	<0.001	0.95 (0.93-0.97)	<0.001
2012-2017	0.87 (0.85-0.89)	<0.001	0.87 (0.85-0.89)	<0.001

### Validation and development of nomograms

**Fig. 3:**
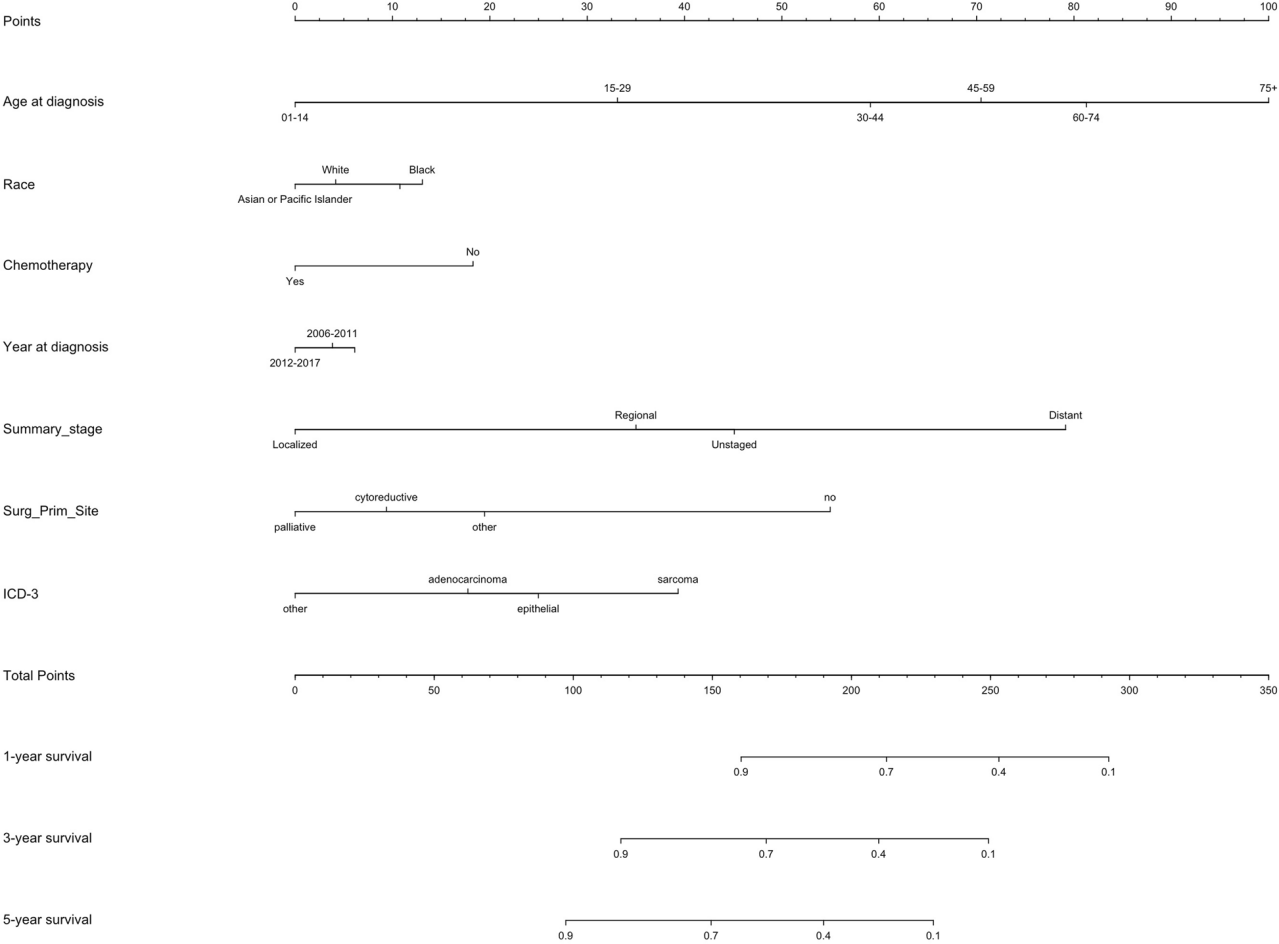
This figure shows a nomogram predicting 12-, 36-, and 60-month OS in patients with ovarian cancer. Points were assigned for OC, age, race, chemotherapy, year of diagnose, summary stage, surgery, and ICD-O-3 by drawing a line upward from the corresponding values to the “points line.” The “total points” are calculated as the sum of the individual score of each of the 7 variables included in the nomogram

**Fig. 4:**
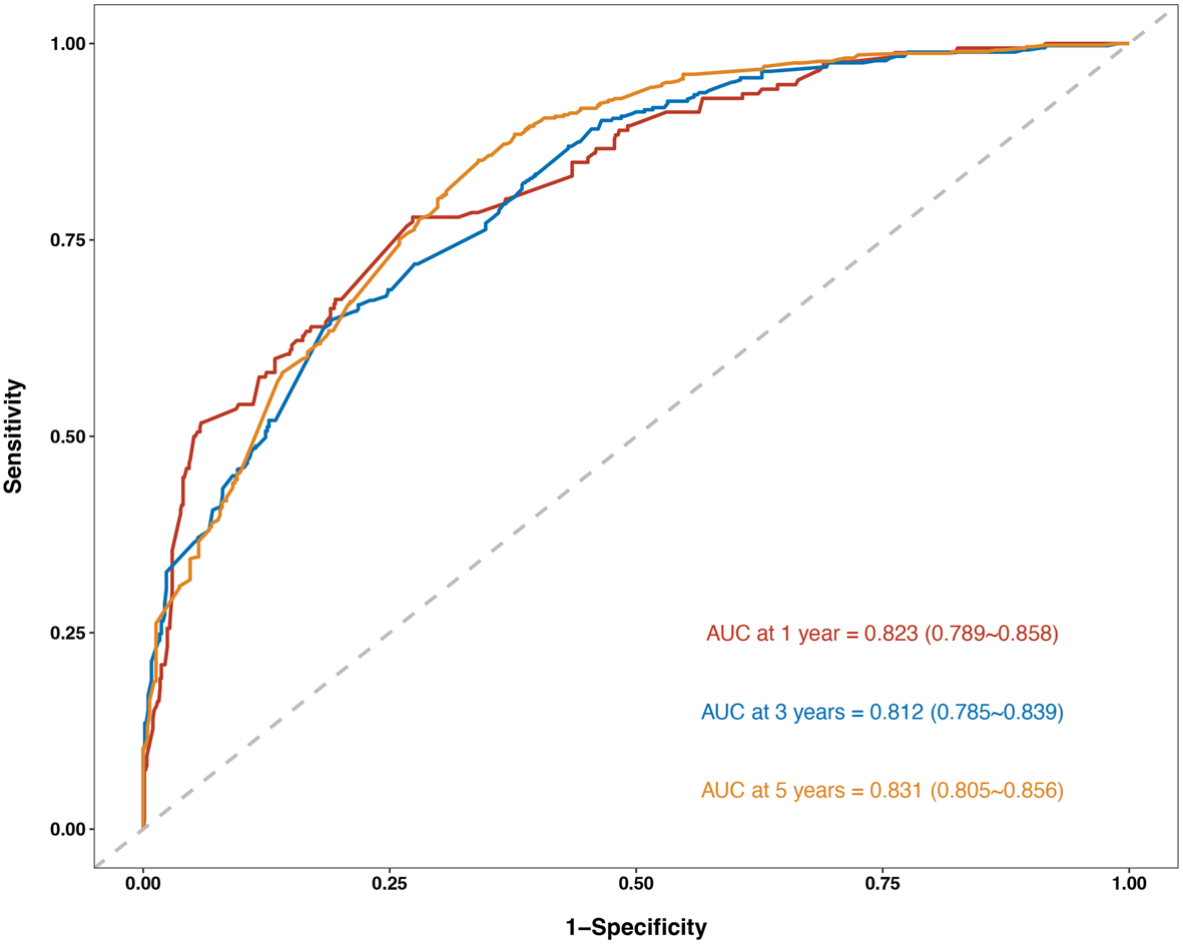
ROC curve comparison between nomogram and independent predictor variables, 12-, 36-, and 60-OS

Based on our previously identified independent prognostic factors, we developed a nomogram to predict 12-, 36-, and 60-month OS in ovarian cancer patients (Fig. 3). We then conducted an effective evaluation of the overall performance of the nomogram, with a C-index of 0.759 (95%CI: 0.757–0.761), which indicates that the model has sufficient discriminative power for prediction (Fig. S5). At the same time, we also used calibration curves to evaluate OS at 12-, 36-, and 60-months (Fig. S6). In addition, the AUC values of the ROC curve at 12-, 36-, and 60-months under our training model are 0.823 (95%CI: 0.789–0.858), 0.812 (95%CI: 0.785–0.839), and 0.831 (95%CI: 0.805–0.856), which means that our model has good discrimination ability (Fig. 4). Based on our above time-dependent ROC curve results, DCA analysis was conducted for 12-, 36-, and 60-month periods, and the results showed that the net benefit of our proposed model increased significantly and had a wider threshold probability range (Fig. S7).

### Stratified prediction ability of nomogram for cardiovascular mortality risk in patients with ovarian cancer

In addition, we used cardiovascular death as the outcome variable to observe the impact of the above independent risk factors on OS of ovarian cancer patients. These independent factors are all statistically significant (*p* < 0.01). More than half of patients with ovarian cancer develop CVD when they are over 60 years old, and patients with adenocarcinoma are more likely to develop CVD (Table S1).

**Fig. 5:**
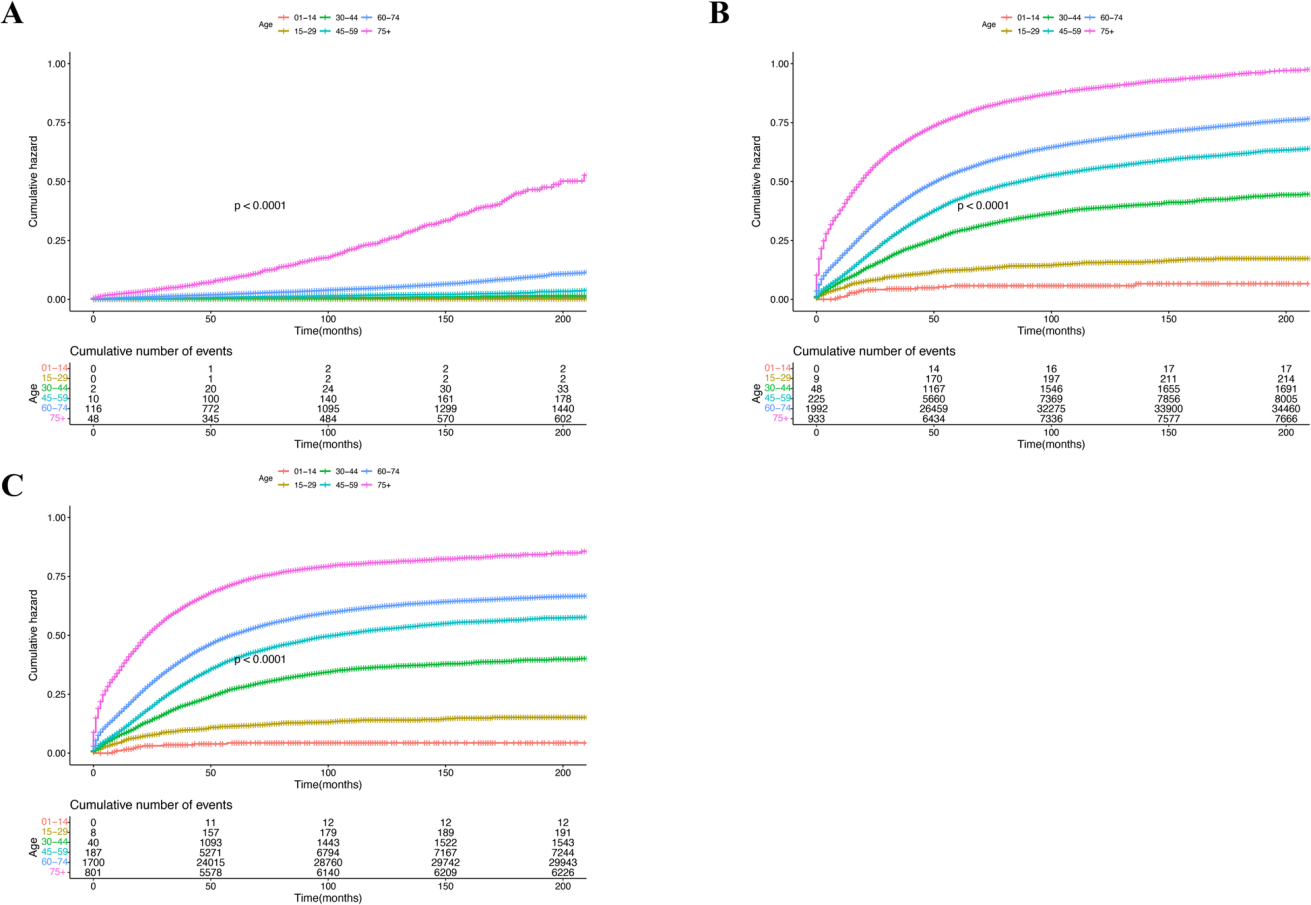
Effects of different age at diagnose on cardiovascular mortality risk (**A**), overall survival time (**B**), and tumor-specific death (**C**) in patients with ovarian cancer

The risk of cardiovascular death in ovarian cancer patients who undergo chemotherapy is significantly lower than that of patients who do not receive chemotherapy. This may be due to systemic treatment that delays the progression of the disease[HR: 0.48 (95%CI:0.44–0.52), *p* < 0.001]( Table S2). Age is still a strong factor affecting cardiovascular death in patients with ovarian cancer. Early intervention and treatment of cardiovascular disease in elderly patients with ovarian cancer will be beneficial to prolonging OS (Fig. 5). Ovarian cancer patients who did not receive chemotherapy were at higher risk of cardiovascular death over time (Fig. S8). Black people have a higher risk of cardiovascular death than White, (American Indian/Alaska Native), and (Asian or Pacific Islander) people (Fig. S9). The results show that even if patients with ovarian cancer undergo palliative surgical treatment, their risk of cardiovascular death is significantly lower than that of patients who do not undergo surgical treatment(Fig. S10). Our statistical analysis shows that patients with ovarian histological type carcinosarcoma have a higher risk of cardiovascular death than those with epithelial tumors(Fig. S11). We included the above independent risk factors to evaluate OCSS. The results show that OCSS is seriously affected in older patients, distant metastasis and no surgery are key factors in their poor prognosis (Fig. S12).

## Discussion

This study is based on the large SEER database, which features a retrospective cohort design and appropriate bias-controlled analysis of correlations among patients with ovarian cancer related to cardiovascular disease deaths. There are many common risk factors between cancer and cardiovascular disease, such as smoking, radiation, air pollution and metabolic syndrome [11]. It has been found that the risk of cardiovascular death in patients with tumors of different origins is vastly different [12–14]. Newly diagnosed other tumors may be a psychological and emotional distress to promote the occurrence of CVM in patients with previously diagnosed ovarian cancer, which is not inconsistent with previous researches [15]. Studies have shown that the presence of diabetes mellitus (DM) in breast, lung, colorectal, and gastric cancers is strongly associated with cardiovascular death in cancer patients [16]. Another study showed that patients with breast, endometrial, and ovarian cancer who received endocrine therapy had significantly increased CVD complications and cardiovascular risk factors (CVRF) [17]. The number of diagnoses of ovarian malignancies of reproductive origin in women has been increasing in recent years, but the number of deaths due to cardiovascular disease has been decreasing year on year, which may be inextricably linked to advances in ovarian cancer treatment strategies and improvements in multiple management modalities. However, the treatment of ovarian cancer is an extremely complex process, with repeated recurrences as well as chemotherapy leading to a significant increase in the risk of cardiovascular disease [18]. Patients with ovarian cancer are at increased risk of developing CVM for a variety of reasons during our cut-off follow-up period. Our study found that age, race, chemotherapy, histological type, summary stage, and surgery were independent predictors of the development of CVM in patients with ovarian cancer.

Nomogram is a visually friendly risk statistical prediction model that can provide better survival risk prediction for clinical patients and patients. Currently, this model has been widely used in a variety of malignant tumors due to its simplicity and reliable predictive capabilities [19, 20]. Meanwhile, through the model we established, we can find that the age at diagnosis is one of the most important factors for the occurrence of CVD in ovarian cancer patients. Therefore, timely prevention and treatment of elderly ovarian cancer patients has obvious benefits in reducing the risk of cardiovascular death [21].

Several past studies have shown that cancer patients are more likely to experience symptoms of anxiety and depression, which to a certain extent increase the cardiovascular load and lead to pathological conditions [6, 22]. Therefore, timely spiritual and psychological support for newly diagnosed cancer patients may delay the progression of the disease to a certain extent. The risk of VTE in cancer patients also affects prognosis and disease progression. The possible reasons are the coagulation cascade and tumor growth, but VTE often appears within a few months or a year after surgical treatment of the tumor [23]. In our data, patients with ovarian cancer were almost always diagnosed after menopause, or entered menopause after surgical treatment. Postmenopausal physical status is more likely to increase the risk of cardiovascular disease death. In clinical decision-making, when OC patients are over 75 years old, in addition to paying attention to the ovarian cancer itself, we should also pay more attention to the risk of CVM in this type of patients and perform timely intervention.

Patients with early-stage ovarian cancer can undergo surgical resection for comprehensive staging. After surgery, the need for adjuvant treatment is determined based on pathological staging and histological grading. Patients with advanced ovarian cancer need to evaluate whether satisfactory tumor reduction surgery can be achieved based on the patient's general condition and CT score [24]. If satisfactory tumor reduction surgery cannot be achieved after comprehensive evaluation, systemic treatment is required first, usually after 2–3 cycles. After systemic treatment, patients were re-evaluated, and intermediate debulking surgery was performed. Systemic treatment was continued after surgery for a total of 6–8 cycles [25]. For those who achieve complete remission or partial remission after systemic treatment, targeted drug maintenance therapy may be considered. First-line systemic therapy for ovarian cancer patients mainly includes platinum-based chemotherapy ± anti-angiogenic drugs or maintenance therapy with poly-adenosine diphosphate ribose polymerase (PARP) inhibitors [26–29]. Platinum drugs are cell cycle non-specific drugs and are alkylating cytotoxic drugs in a broad sense. They mainly form Pt–DNA adducts with DNA after entering tumor cells, inhibit DNA replication and transcription, thereby mediating tumor cell necrosis or apoptosis. This will also cause DNA damage to normal cells, such as cardiomyocytes, vascular endothelial cells, etc. When these repair systems are mutated, it will increase the risk of cardiovascular-related toxicity during anti-tumor treatment and cause cardiovascular disease-related death [30]. Meanwhile, genetically related heart diseases also require our vigilance. They will not only promote the occurrence and progression of tumors, but also cause heart-related toxic effects during anti-tumor systemic treatments [31, 32].

In this study, we still have some shortcomings, such as income level, education, marital status, the presence of other malignant tumors, specific chemotherapy regimens, secondary cytoreductive surgery, smoking, drinking and other variables have not been included, and these are highly likely to affect the risk of cardiovascular death in patients with ovarian cancer. This is likely to indicate that the risk of CVM in patients with ovarian cancer is underestimated, and we need to conduct more in-depth population studies.

## Competing interests

The authors declare that the research was conducted in the absence of any commercial or financial relationships that could be construed as a potential conflict of interest.

### Supplementary Information

**Additional File 1.**

**Additional File 2.**

**Additional File 3. Table S1.** Baseline characteristics of CVD in patients with ovarian cancer. **Table S2.** Competing risk regression analysis for predictors of cardiovascular mortality in patients with ovarian cancer.

## Data Availability

No datasets were generated or analysed during the current study.

## Abbreviations

OC: Ovarian cancer
CVM: Cardiovascular mortality
CVD: Cardiovascular death
SEER: Surveillance Epidemiology, and end results
OS: Overall survival
OCSS: Ovarian cancer‐specific survival
ICD-O-3: International classification of diseases for oncology, 3rd edition
ROC: Receiver operation characteristic curve
AUC: Area under curve
DCA: Decision curve analysis
95% CI: 95% Confidence interval
CVRF: Cardiovascular risk factors
PARP: Poly ADP-ribose polymerase
VTE: Venous thromboembolism
