# Supplementary material for: Cardiovascular mortality risk in patients with ovarian cancer: a population-based study
Source: J Ovarian Res. 2024 Apr 25;17:88. doi: 10.1186/s13048-024-01413-4 (PMC11044491; doi:10.1186/s13048-024-01413-4)
Supplement: Supplementary file 1 — Additional File 1. [file 13048_2024_1413_MOESM1_ESM.docx]

| 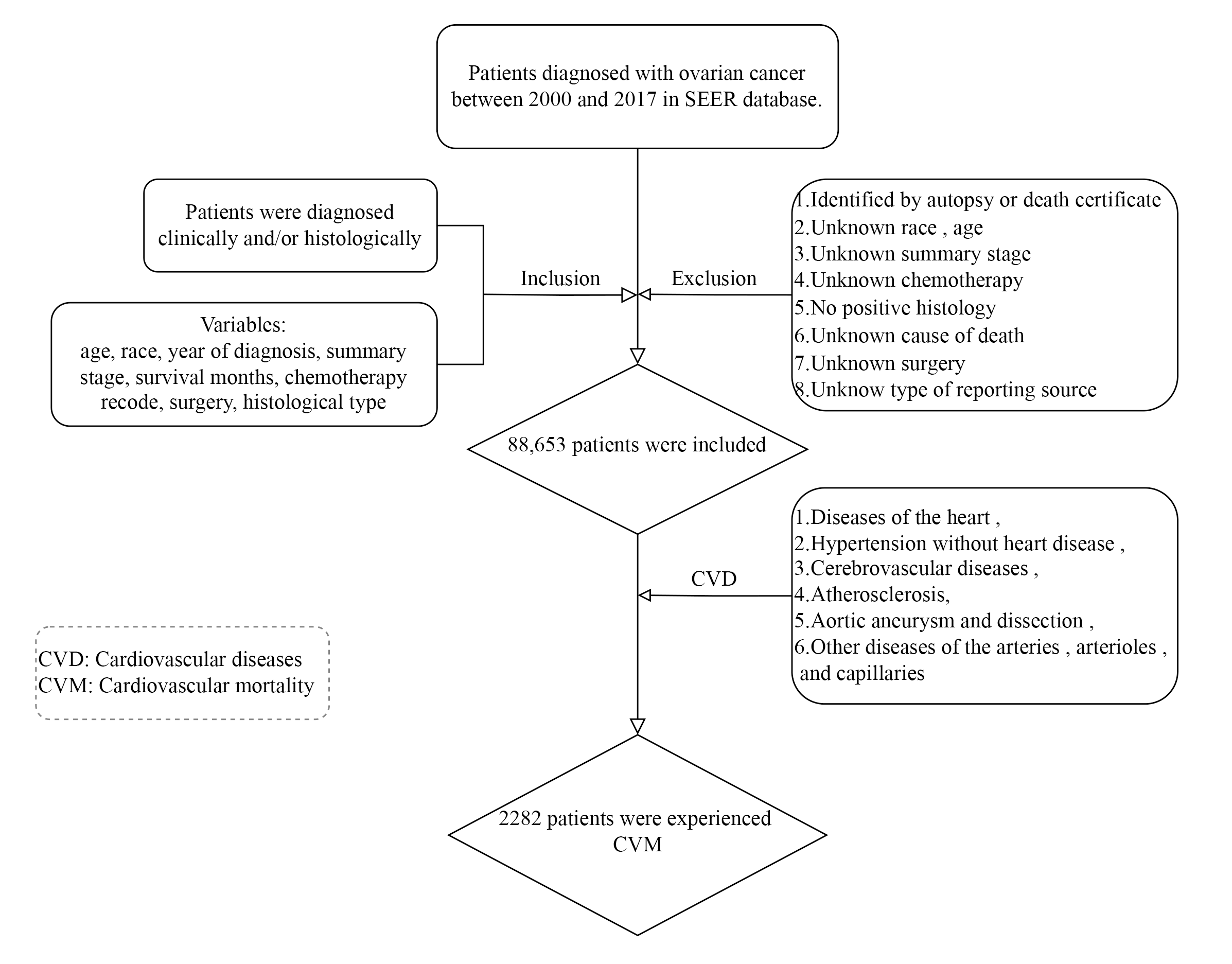 |
| --- |

FigureS1: Overall design of the study and inclusion criteria.

| 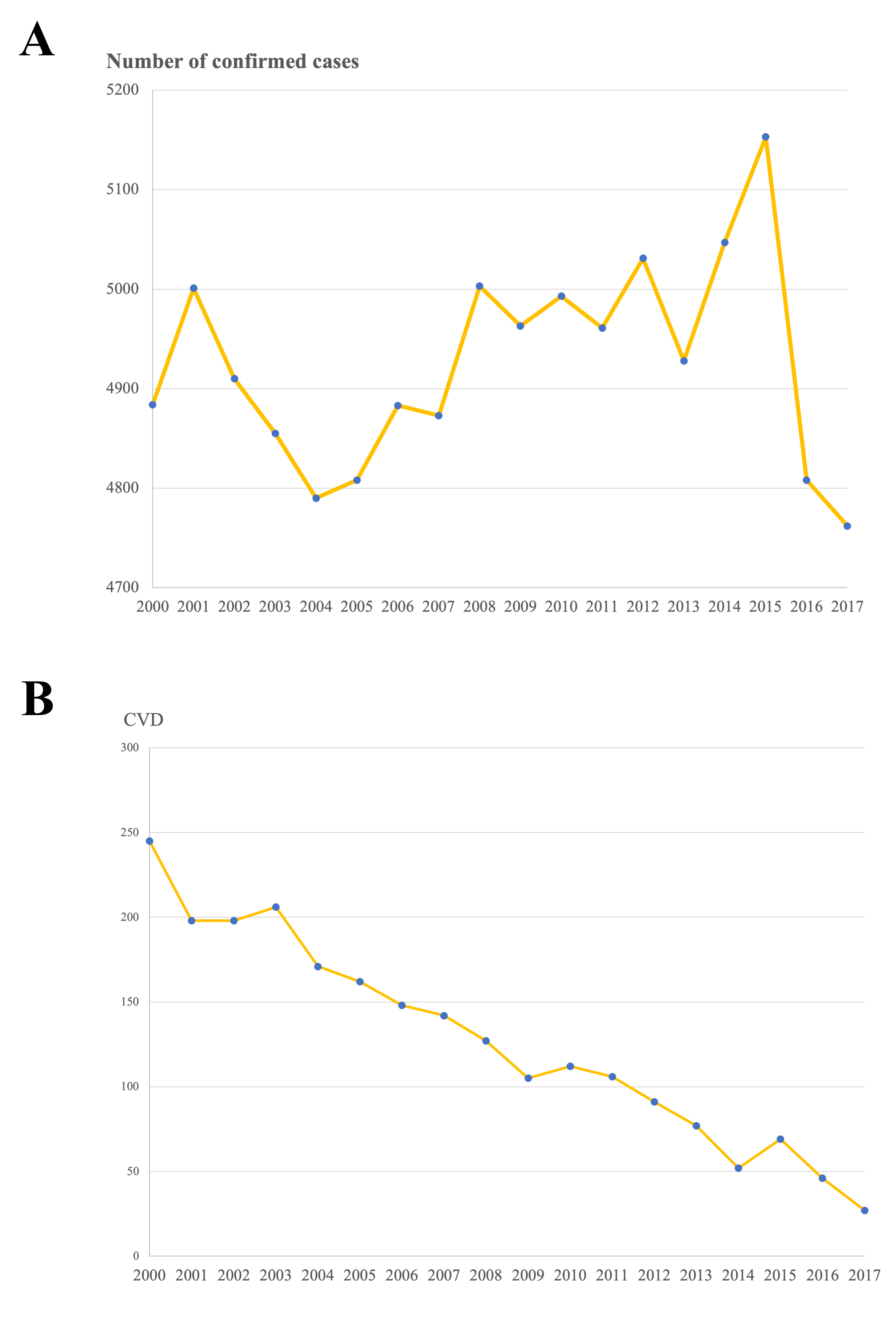 |
| --- |

FigureS2：Changes in the number of ovarian cancer diagnoses over time(A). Changes in the number of CVD deaths over time in patients with ovarian cancer(B).

**
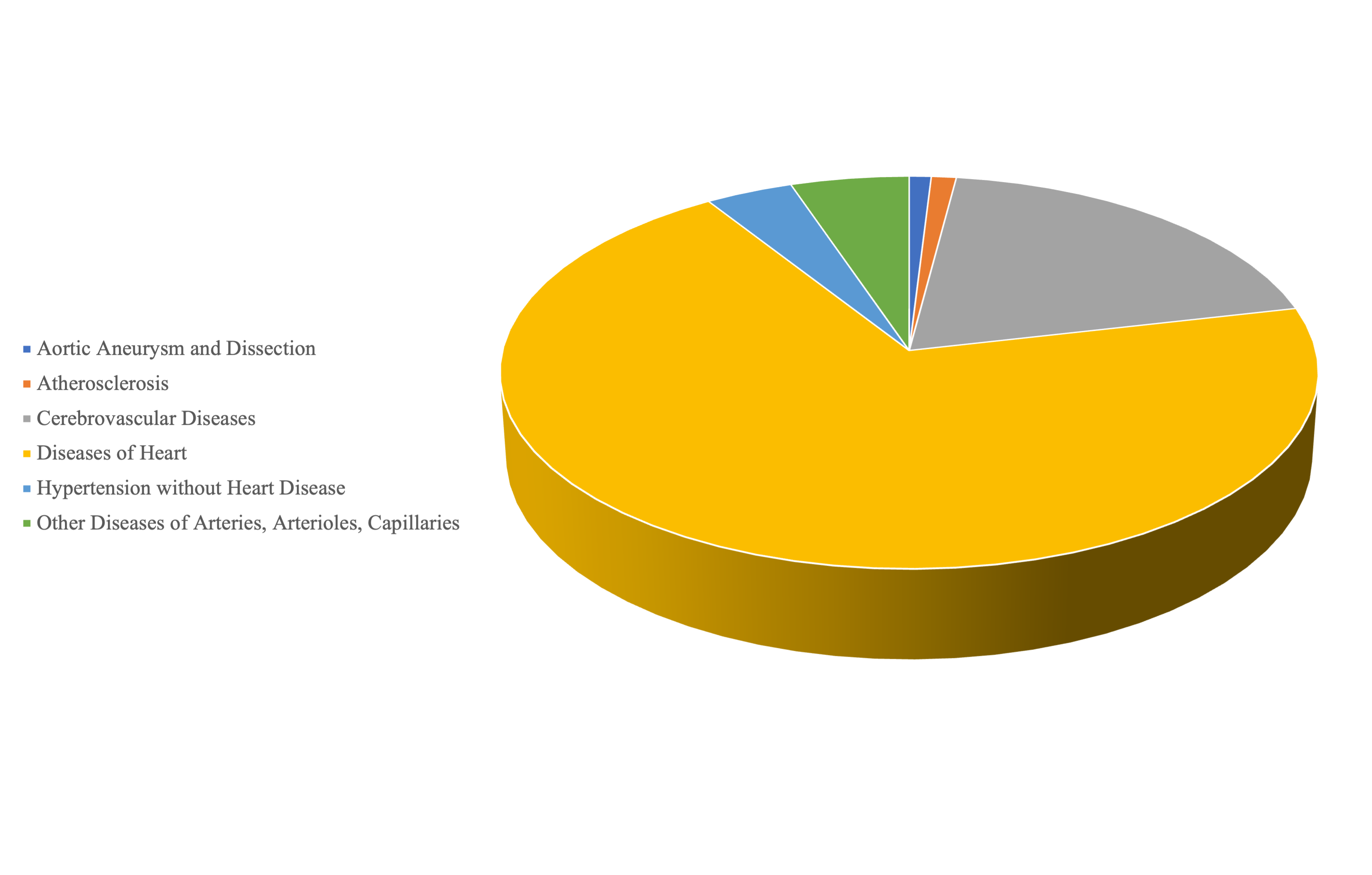
**

FigureS3：Proportions of different types of CVD, with disease of heart accounting for the highest proportion.


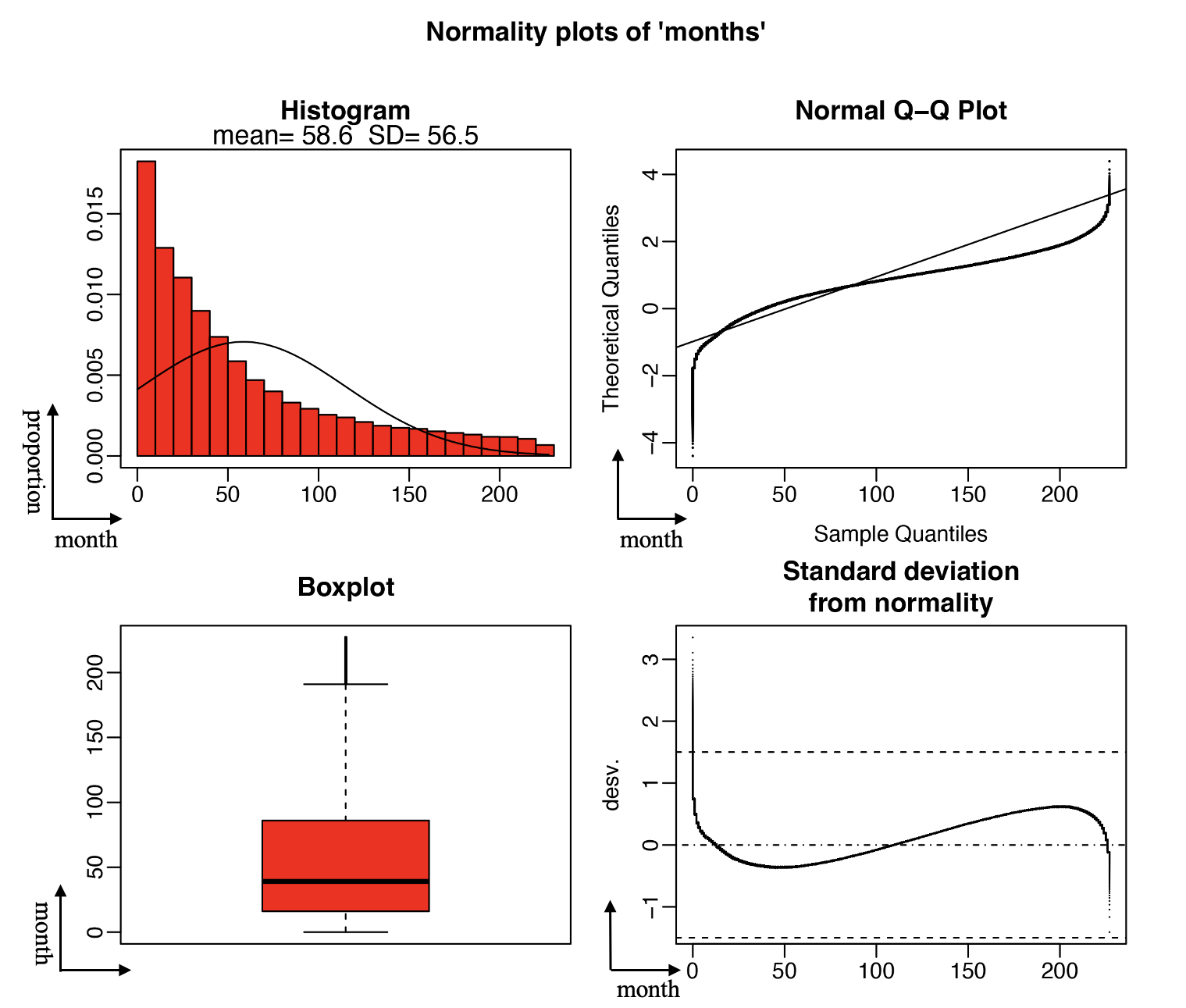


FigureS4：Normal distribution plot of overall survival time.

|  |
| --- |

FigureS5: Change curve of C-index [0.759 (95%CI: 0.757-0.761)] with predicted overall survival time.

| \| 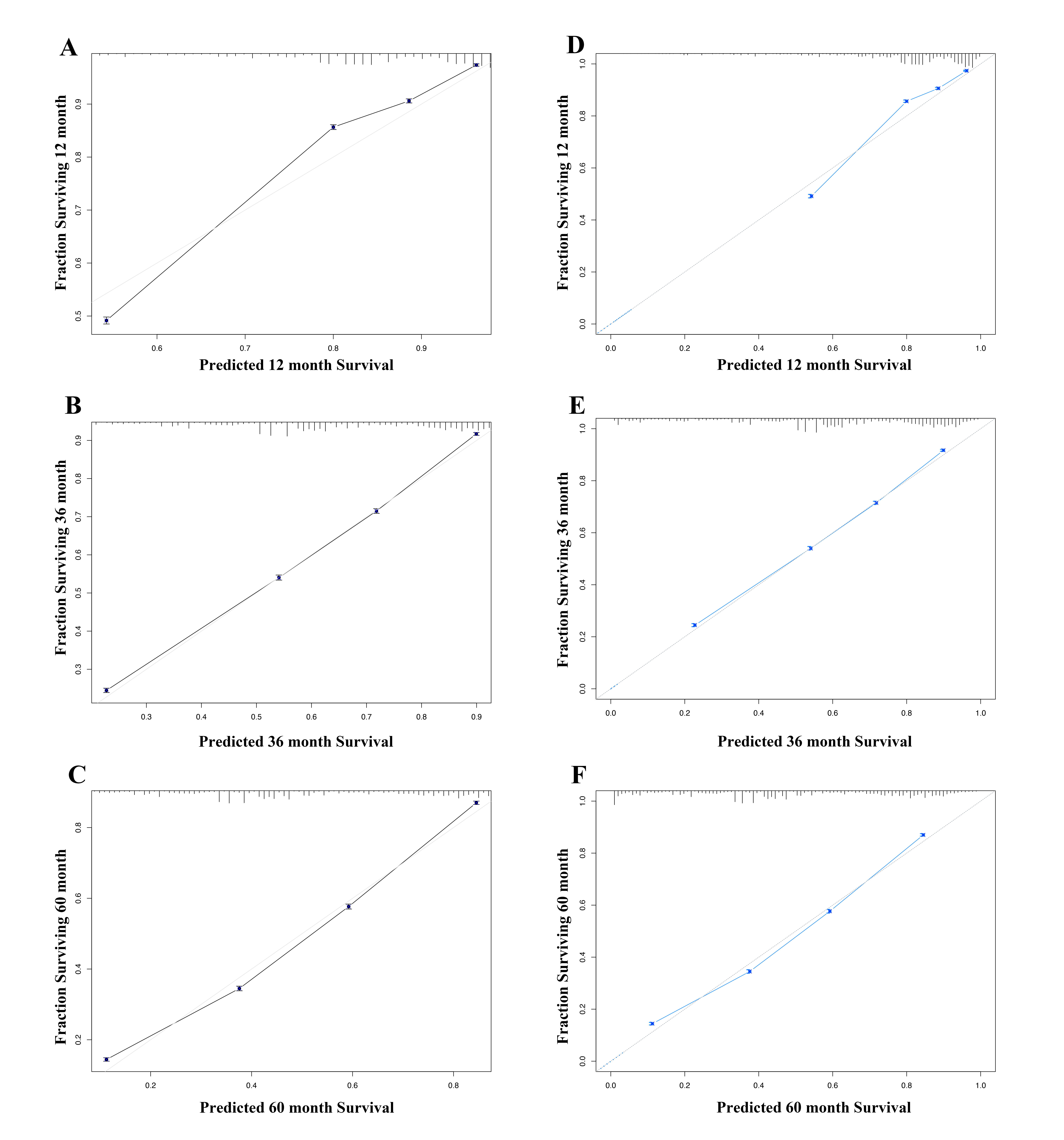 \| \| --- \| |
| --- | --- |

FigureS6: 12-(A, D), 36-(B, E) and 60-(C, F) OS. The dashed line represents a perfect match between the nomogram prediction (X-axis) and the actual survival outcome (Y-axis). The closer the distance from the point to the dashed line, the higher the prediction accuracy.

| 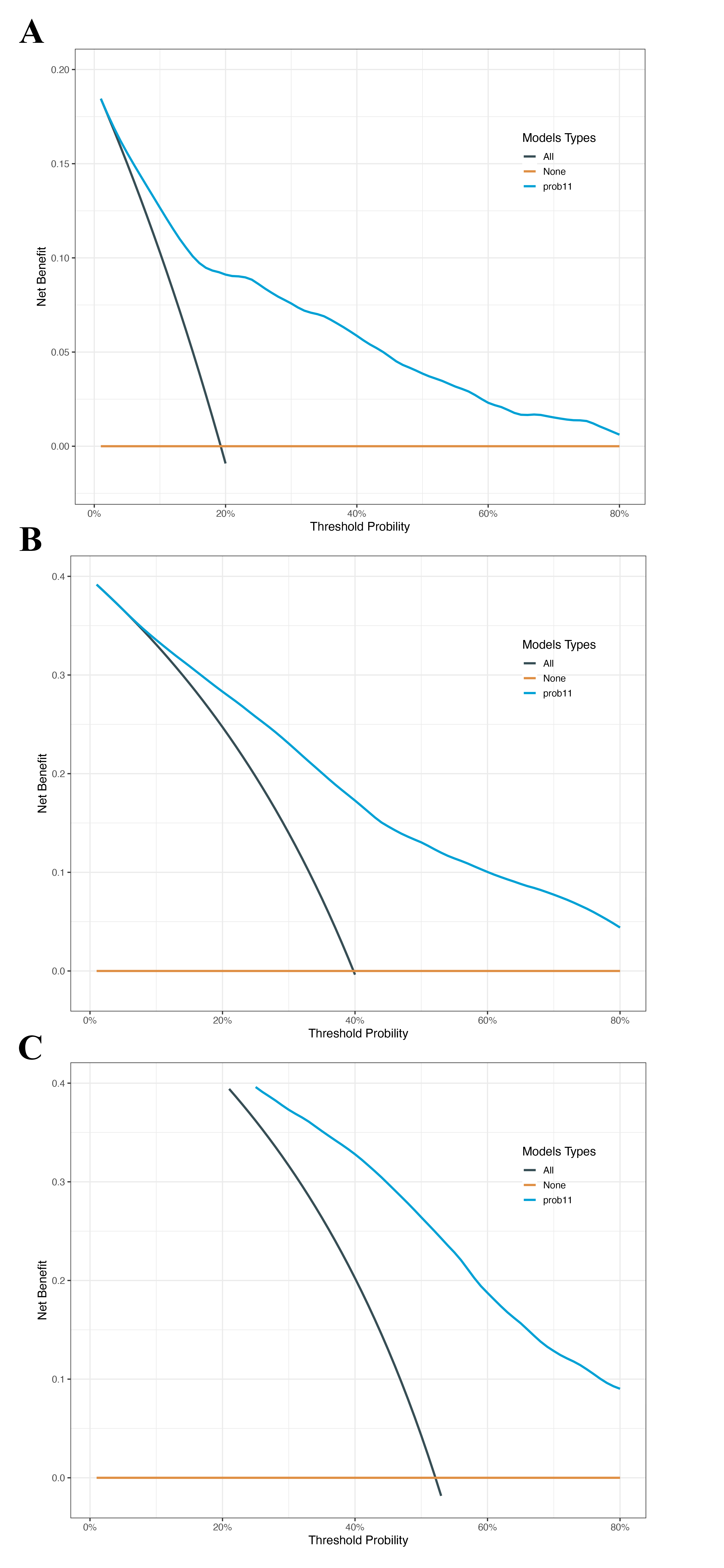 |
| --- |

FigureS7: Based on the model, DCA curve points predict and verify 12-(A), 36-(B) and 60-(C)OS.

| 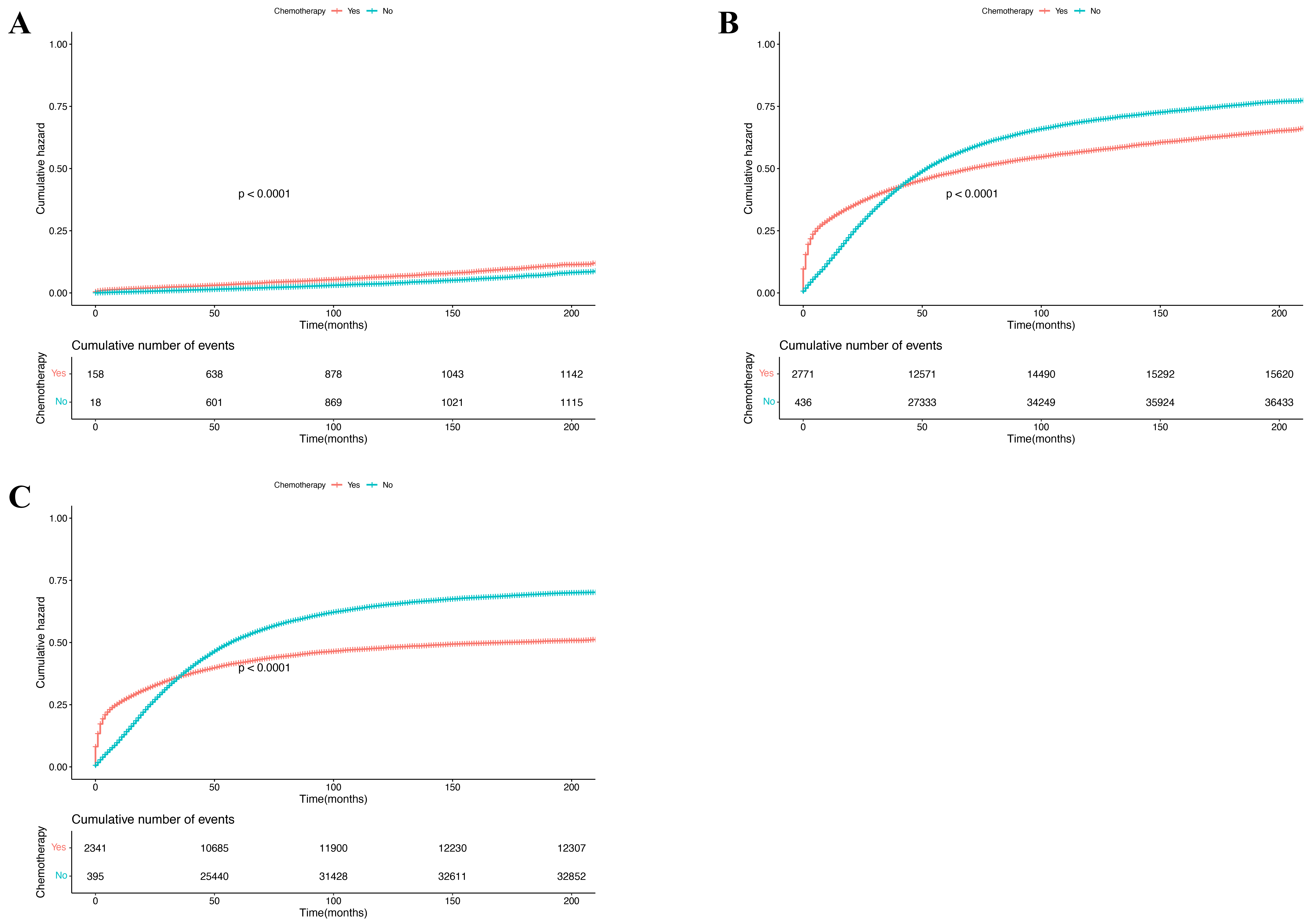 |
| --- |

FigureS8: Whether chemotherapy affects cardiovascular mortality risk, overall survival time, and tumor-specific death in patients with ovarian cancer.

| 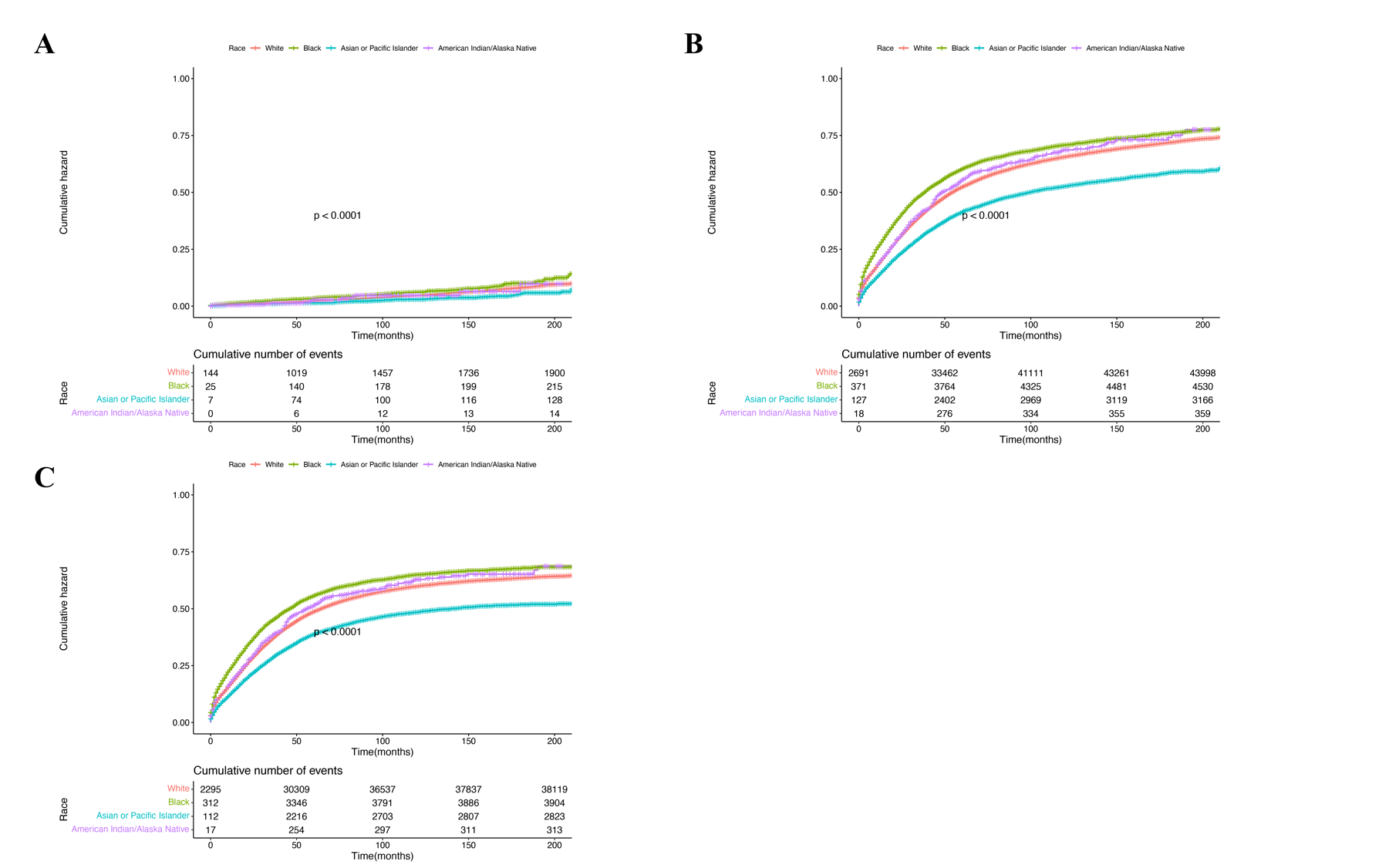 |
| --- |

FigureS9: Effects of different race on cardiovascular mortality risk(A), overall survival time(B), and tumor-specific death(C) in patients with ovarian cancer.

| **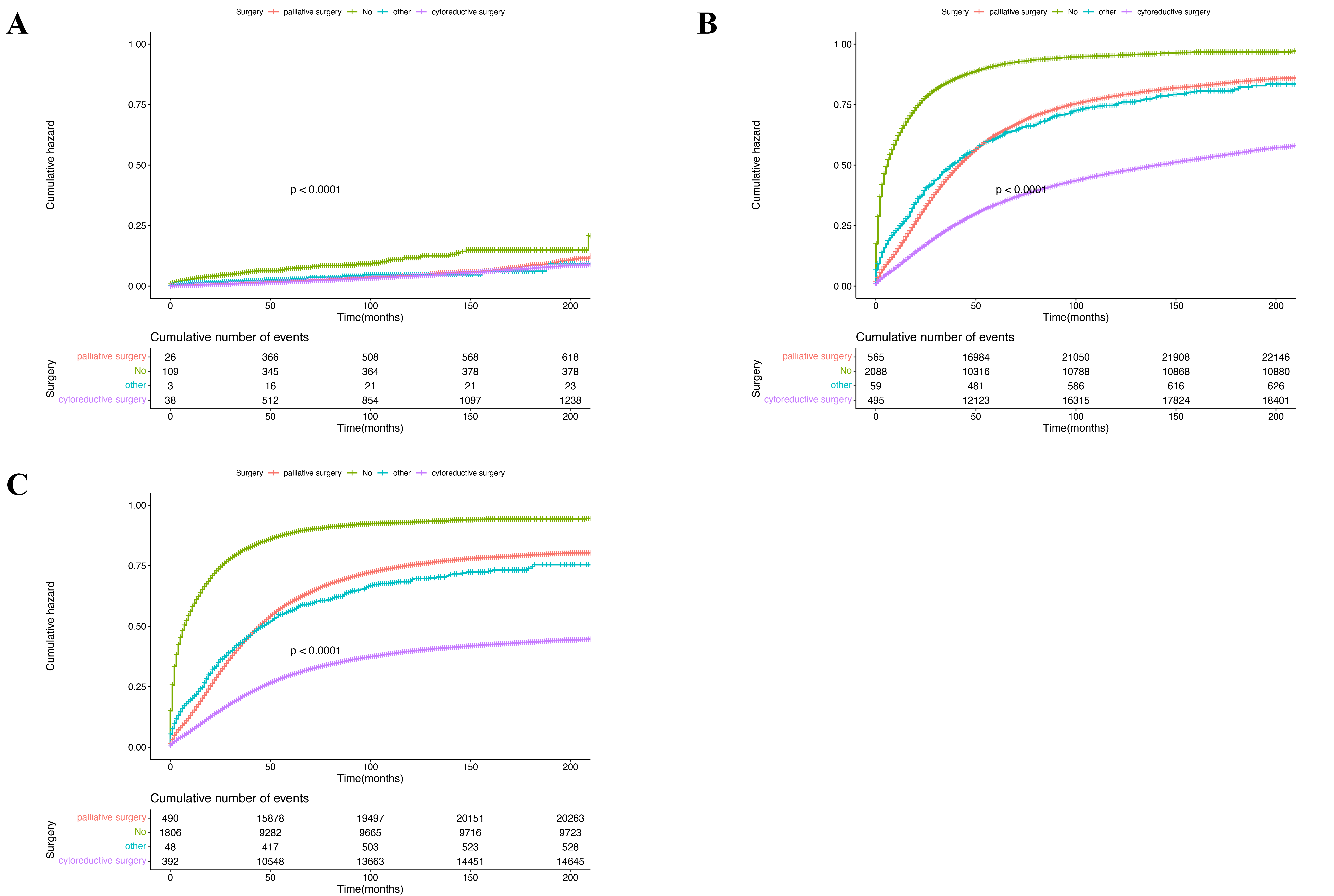** |
| --- |

FigureS10: Effects of different surgical methods on cardiovascular mortality risk(A), overall survival time(B), and tumor-specific death(C) in patients with ovarian cancer.

| 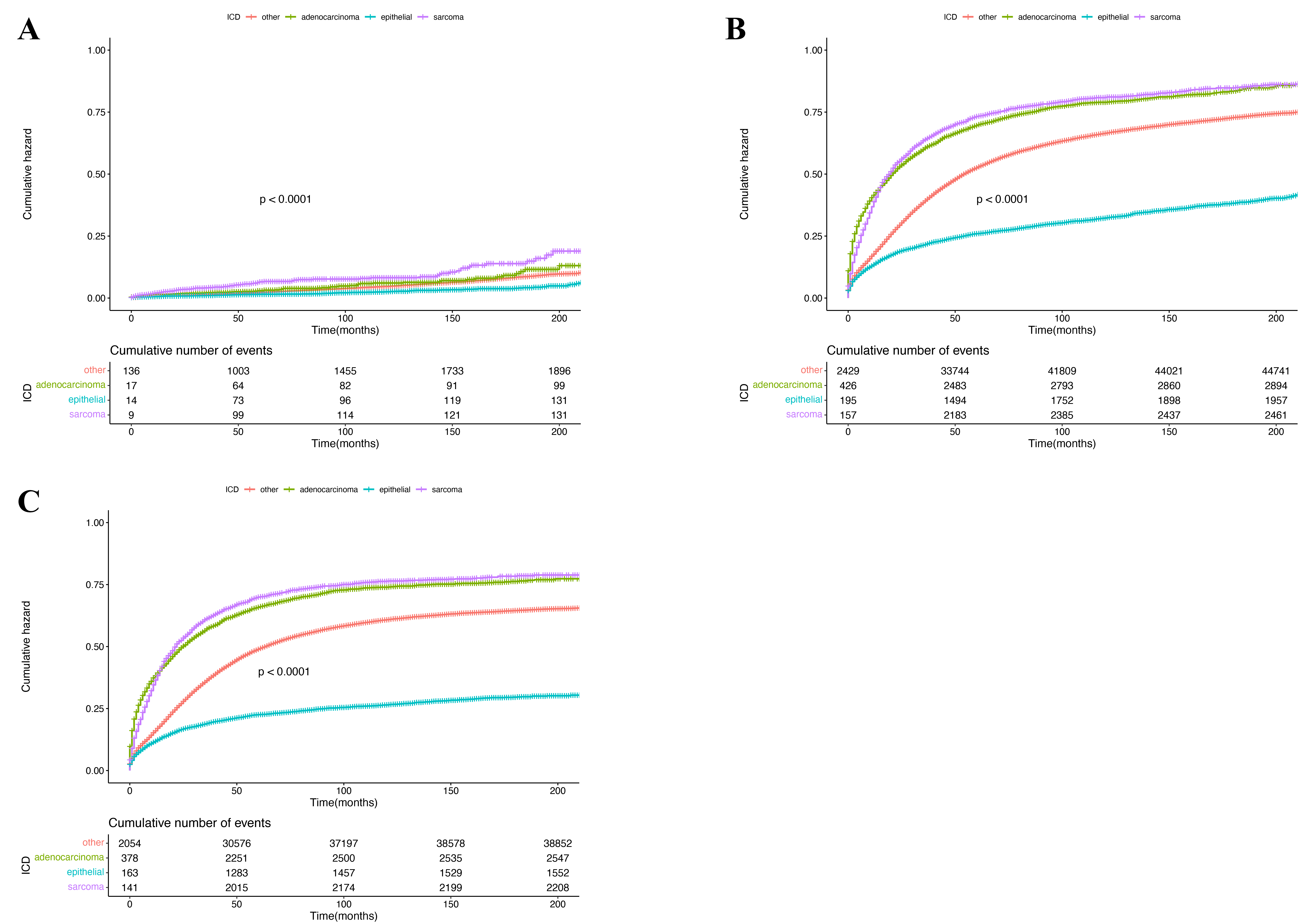 |
| --- |

FigureS11: Effects of different ICD-O-3 on cardiovascular mortality risk(A), overall survival time(B), and tumor-specific death(C) in patients with ovarian cancer.

| 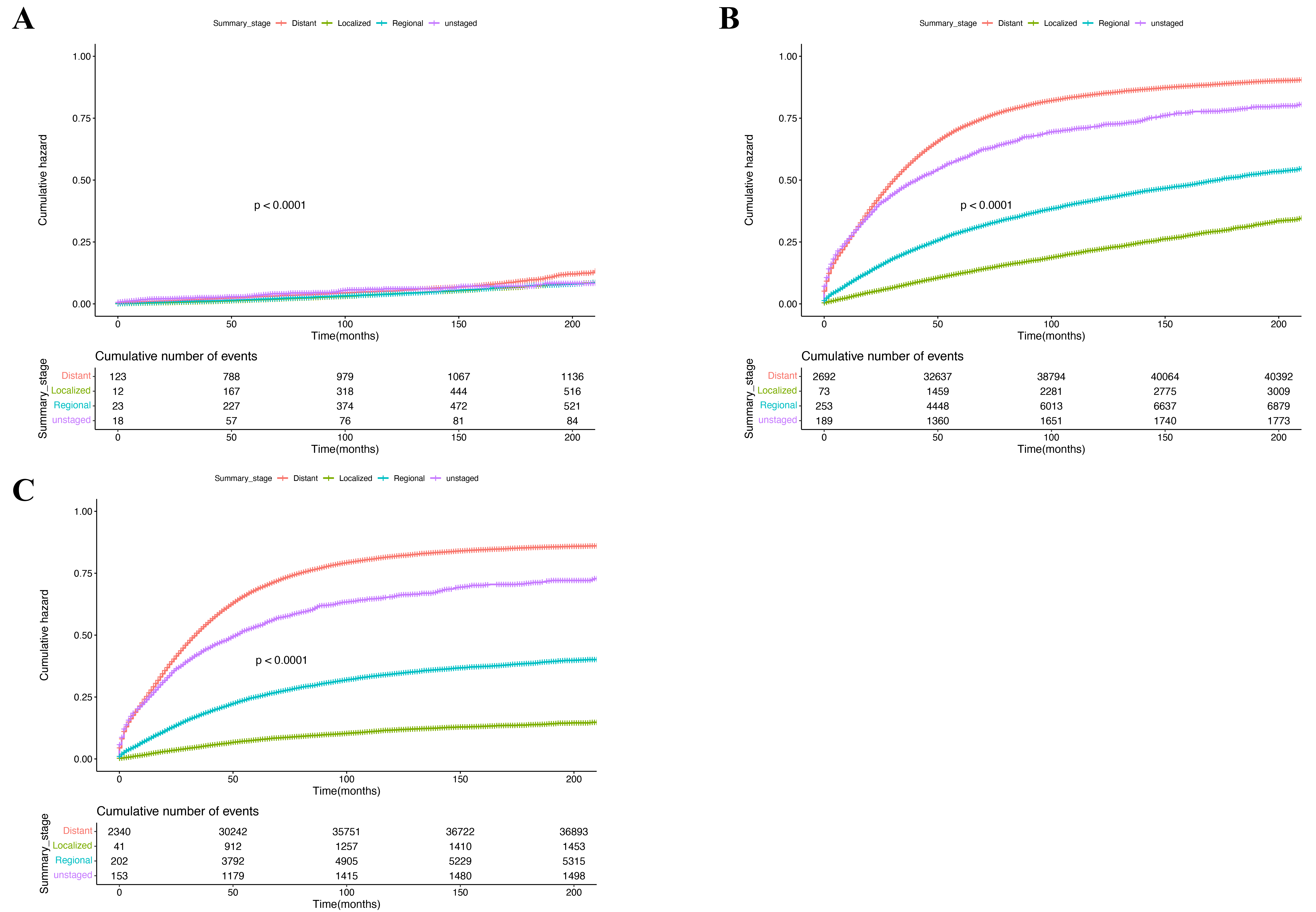 |
| --- |

FigureS12: Effects of different stages on cardiovascular mortality risk(A), overall survival time(B), and tumor-specific death(C) in patients with ovarian cancer.

|  |
| --- |
