# Supplementary material for: Cardiovascular mortality risk in patients with ovarian cancer: a population-based study
Source: J Ovarian Res. 2024 Apr 25;17:88. doi: 10.1186/s13048-024-01413-4 (PMC11044491; doi:10.1186/s13048-024-01413-4)
Supplement: Supplementary file 2 — Additional File 2. [file 13048_2024_1413_MOESM2_ESM.rtf]

c1 <- makeCluster(12)library(rms)library(foreign)library(survival)library(riskRegression)library(timeROC)library(pROC)library(pec)library(ggplot2)library(caret)library(autoReg)library(dplyr)library(nomogramFormula)options(rgl.useNULL = TRUE)library(export)library(survminer)library(moonBook)library(compareGroups)cGroupsWUI(port=8102L)setwd()rt<-read.csv('test1.CSV')str(rt)for (i in names(rt)[c(2:8)]){rt[,i] <- as.factor(rt[,i])}str(rt)dd <- datadist(rt)option<-options(datadist = "dd")units(rt$months)<-"month"names(rt)options(scipen=100) options(digits=3) fit<-coxph(Surv(months,os)~Age+Race+Chemotherapy+Summary_stage+             Surg_Prim_Site+ICD+Year,data=rt)result<-autoReg(fit,uni=T,multi=T,final=T,digits=2,threshold=0.05)resultwrite.csv(result,"1.csv")coxm<-cph(Surv(months,os)~Age+Race+Chemotherapy+Summary_stage+            Surg_Prim_Site+ICD+Year,x=T,y=T,surv=T,data=rt,time.inc=12)surv<-Survival(coxm)sur_1_year<-function(x)surv(12,lp=x)sur_3_year<-function(x)surv(36,lp=x)sur_5_year<-function(x)surv(60,lp=x)nom<-nomogram(coxm,fun=list(sur_1_year,sur_3_year,sur_5_year),lp=T,              funlabel=c('1-year Survival','3-year Survival','5-year Survival'),              maxscale=100,              fun.at=c('0.9','0.8','0.7','0.6','0.5','0.4','0.3','0.2','0.1'))print(nom)results<-formula_lp(nomogram=nom)points<-points_cal(formula=results$formula,lp=coxm$linear.predictors)results<-formula_rd(nomogram=nom)rt$points<-points_cal(formula=results$formula,rd=rt)rt$linear.predictors<-coxm$linear.predictorsrt$survprob1<-predictSurvProb(coxm,newdata=rt,times=c(12,36,60))write.csv(rt,file='points_os.csv',row.names=F)hist(rt$points)rt<-read.csv('test1.CSV')str(rt)rt$Age<-factor(rt$Age,labels=c("01-14","15-29","30-44","45-59","60-74","75+"))rt$Race<-factor(rt$Race,labels=c("White","Black","Asian or Pacific Islander","American Indian/Alaska Native"))rt$Chemotherapy<-factor(rt$Chemotherapy,labels=c("No","Yes"))rt$Summary_stage<-factor(rt$Summary_stage,labels=c("Distant","Localized","Regional","Unstaged"))rt$Surg_Prim_Site<-factor(rt$Surg_Prim_Site,labels=c("cytoreductive","no","other","palliative"))rt$ICD<-factor(rt$ICD,labels=c("adenocarcinoma","epithelial","other","sarcoma"))rt$Year<-factor(rt$Year,labels=c("2000-2005","2006-2011","2012-2017"))str(rt)label(rt$Age)<-"Age at diagnosis"label(rt$Race) <-"Race"label(rt$Chemotherapy)<-"Chemotherapy"label(rt$Summary_stage)<-"Summary_stage"label(rt$Surg_Prim_Site)<-"Surg_Prim_Site"label(rt$ICD)<-"ICD-3"label(rt$Year)<-"Year at diagnosis"dd<-datadist(rt)option<-options(datadist="dd")f<-cph(Surv(months,os)~Age+Race+Chemotherapy+Year+Summary_stage+         Surg_Prim_Site+ICD,x=T,y=T,surv=T,data=rt,time.inc=12)summary(f)surv<-Survival(f)nom<-nomogram(f,fun=list(function(x)surv(12,x),function(x)surv(36,x),function(x)surv(60,x)),               lp=F,funlabel=c("1-year survival","3-year survival","5-year survival"),               maxscale=100,               fun.at=c(0.9,0.7,0.4,0.1))plot(nom,xfrac=0.3,#     cex.axis=0.7,#     cex.var=0.9,#     points.label='Points',total.points.label='Total Points')print(nom)#res.cox1<-coxph(Surv(months,os)~Age+Race+Chemotherapy+Year+Summary_stage+                  Surg_Prim_Site+ICD,data=rt)res.cox1summary(res.cox1)#rcorrcens(Surv(months,os)~predict(f),data=rt)sum.surv<-summary(coxph(Surv(months,os)~Age+Race+Chemotherapy+Year+Summary_stage+                          Surg_Prim_Site+ICD,data=rt))cindex<-sum.surv$concordancecindex##model1<-cph(Surv(months,os)~Age+Race+Chemotherapy+Year+Summary_stage+              Surg_Prim_Site+ICD,x=T,y=T,surv=T,data=rt)#A1<-pec::cindex(list("model1"=model1),                formula=Surv(months,os)~Age+Race+Chemotherapy+Year+Summary_stage+                  Surg_Prim_Site+ICD,data=rt,                eval.times=seq(0,200,1))#plot(A1,     col=c("red"),#     xlab="time (month)",#     ylab="Concordance index",     ylim = c(0.4,1),#     xlim = c(0,200),     legend.x=100,#     legend.y=1,      legend.cex=1#);title(main="time C-index")#units(rt$months)<-"month"f<-cph(Surv(months,os)~Age+Race+Chemotherapy+Year+Summary_stage+         Surg_Prim_Site+ICD,x=T,y=T,surv=T,data=rt,time.inc=12)cal<-calibrate(f,cmethod="KM",method="boot",u=12,m=22000,B=100)#plot(cal)plot(cal,xlim=c(0,1.0),ylim=c(0,1.0),lwd=2,lty=2,errbar.col=c(rgb(30,144,255,maxColorValue=255)),col=c(rgb(30,144,255,maxColorValue=255)))lines(cal,lwd=2,lty=3,col=c(rgb(30,144,255,maxColorValue=255)))abline(0,1,lty=3,lwd=1,col=c(rgb(112,128,144,maxColorValue=255)))#f<-cph(Surv(months,os)~Age+Race+Chemotherapy+Year+Summary_stage+         Surg_Prim_Site+ICD,x=T,y=T,surv=T,data=rt,time.inc=36)cal<-calibrate(f,cmethod="KM",method="boot",u=36,m=22000,B=100)plot(cal)plot(cal,xlim=c(0,1.0),ylim=c(0,1.0),lwd=2,lty=2,errbar.col=c(rgb(30,144,255,maxColorValue=255)),col=c(rgb(30,144,255,maxColorValue=255)))lines(cal,lwd=2,lty=3,col=c(rgb(30,144,255,maxColorValue=255)))abline(0,1,lty=3,lwd=1,col=c(rgb(112,128,144,maxColorValue=255)))#f<-cph(Surv(months,os)~Age+Race+Chemotherapy+Year+Summary_stage+         Surg_Prim_Site+ICD,x=T,y=T,surv=T,data=rt,time.inc=60)cal<-calibrate(f,cmethod="KM",method="boot",u=60,m=22000,B=100)plot(cal)plot(cal,xlim=c(0,1.0),ylim=c(0,1.0),lwd=2,lty=2,errbar.col=c(rgb(30,144,255,maxColorValue=255)),col=c(rgb(30,144,255,maxColorValue=255)))lines(cal,lwd=2,lty=3,col=c(rgb(30,144,255,maxColorValue=255)))abline(0,1,lty=3,lwd=1,col=c(rgb(112,128,144,maxColorValue=255)))#source("stdca.R") library(survival)library(tidyr)library(ggplot2)library(ggsci)units(rt$months)<-"month"Srv=Surv(rt$months,rt$os)coxmod1<-coxph(Srv~Age+Race+Chemotherapy+Year+Summary_stage+                 Surg_Prim_Site+ICD,data=rt)#rt$prob11<-c(1-(summary(survfit(coxmod1,newdata=rt),times=12)$surv))stdca(data=rt,outcome="os",ttoutcome="months",timepoint=12,      predictors=c("prob11"),xstop=0.8,ymin=-0.02,smooth=T)#cox_dca<-stdca(data=rt,outcome="os",ttoutcome="months",timepoint=12,               predictors=c("prob11"),xstop=0.8,ymin=-0.02,smooth=T)cox_dca_df<-cox_dca$net.benefit %>%   pivot_longer(cols=c(all,none,contains("sm")),names_to="models",               values_to="net_benefit")ggplot(cox_dca_df,aes(x=threshold,y=net_benefit))+  geom_line(aes(color=models),linewidth=1.2)+  scale_color_jama(name="Models Types",                   labels=c("All","None","prob11"))+  scale_x_continuous(labels=scales::label_percent(accuracy=1),                     name="Threshold Probility")+  scale_y_continuous(limits=c(-0.02,0.2),name="Net Benefit")+  theme_bw(base_size=14)+  theme(legend.background=element_blank(),        legend.position=c(0.85,0.75))#rt$prob11<-c(1-(summary(survfit(coxmod1,newdata=rt),times=36)$surv))stdca(data=rt,outcome="os",ttoutcome="months",timepoint=36,      predictors=c("prob11"),xstop=0.8,ymin=-0.02,smooth=T)#cox_dca<-stdca(data=rt,outcome="os",ttoutcome="months",timepoint=36,               predictors=c("prob11"),xstop=0.8,ymin=-0.02,smooth=T)cox_dca_df<-cox_dca$net.benefit %>%   pivot_longer(cols=c(all,none,contains("sm")),names_to="models",               values_to="net_benefit")ggplot(cox_dca_df,aes(x=threshold,y=net_benefit))+  geom_line(aes(color=models),linewidth=1.2)+  scale_color_jama(name="Models Types",                   labels=c("All","None","prob11"))+  scale_x_continuous(labels=scales::label_percent(accuracy=1),                     name="Threshold Probility")+  scale_y_continuous(limits=c(-0.02,0.4),name="Net Benefit")+  theme_bw(base_size=14)+  theme(legend.background=element_blank(),        legend.position=c(0.85,0.75))#rt$prob11<-c(1-(summary(survfit(coxmod1,newdata=rt),times=60)$surv))stdca(data=rt,outcome="os",ttoutcome="months",timepoint=60,      predictors=c("prob11"),xstop=0.8,ymin=-0.02,smooth=T)#cox_dca<-stdca(data=rt,outcome="os",ttoutcome="months",timepoint=60,               predictors=c("prob11"),xstop=0.8,ymin=-0.02,smooth=T)cox_dca_df<-cox_dca$net.benefit %>%   pivot_longer(cols=c(all,none,contains("sm")),names_to="models",               values_to="net_benefit")ggplot(cox_dca_df,aes(x=threshold,y=net_benefit))+  geom_line(aes(color=models),linewidth=1.2)+  scale_color_jama(name="Models Types",                   labels=c("All","None","prob11"))+  scale_x_continuous(labels=scales::label_percent(accuracy=1),                     name="Threshold Probility")+  scale_y_continuous(limits=c(-0.02,0.4),name="Net Benefit")+  theme_bw(base_size=14)+  theme(legend.background=element_blank(),        legend.position=c(0.85,0.75))#units(rt$months)<-"month"#f<-cph(Surv(months,os)~Age+Race+Chemotherapy+Year+Summary_stage+         Surg_Prim_Site+ICD,x=T,y=T,surv=T,data=rt,time.inc=12)pred_f_training<-predict(f,rt,type="lp")data_table<-data.frame(time=rt[,"months"],status=rt[,"os"],score=pred_f_training)time_roc_res<-timeROC(T=data_table$time[1:1000],#time                      delta=data_table$status[1:1000],#status                      marker=data_table$score[1:1000],#score                      cause=1,weighting="marginal",#uses the Kaplan-Meier                      times=c(12,36,60),ROC=T,iid=T)time_ROC_df<-data.frame(  TP_1year=time_roc_res$TP[,1],  FP_1year=time_roc_res$FP[,1],  TP_3year=time_roc_res$TP[,2],  FP_3year=time_roc_res$FP[,2],  TP_5year=time_roc_res$TP[,3],  FP_5year=time_roc_res$FP[,3])ggplot(data=time_ROC_df)+  geom_line(aes(x=FP_1year,y=TP_1year),size=1,color="#BC3C29FF")+  geom_line(aes(x=FP_3year,y=TP_3year),size=1,color="#0072B5FF")+  geom_line(aes(x=FP_5year,y=TP_5year),size=1,color="#E18727FF")+  geom_abline(slope=1,intercept=0,color="grey",size=1,linetype=2)+  theme_bw()+  theme(panel.grid=element_blank())+  annotate("text",x=0.75,y=0.25,size=4.5,           label=paste0("AUC at 1 year = ",sprintf("%.3f",time_roc_res$AUC[[1]])),color="#BC3C29FF")+  annotate("text",x=0.75,y=0.15,size=4.5,           label=paste0("AUC at 3 years = ",sprintf("%.3f",time_roc_res$AUC[[2]])),color="#0072B5FF")+  annotate("text",x=0.75,y=0.05,size=4.5,           label=paste0("AUC at 5 years = ",sprintf("%.3f",time_roc_res$AUC[[3]])),color="#E18727FF")+  labs(x="1-Specificity",y="Sensitivity")+  theme(axis.text=element_text(face="bold",size=11,color="black"),        axis.title.x=element_text(face="bold",size=14,color="black",margin=margin(c(15,0,0,0))),        axis.title.y=element_text(face="bold",size=14,color="black",margin=margin(c(0,15,0,0))))time_roc_res$AUCconfint(time_roc_res,level=0.95)$CI_AUC#ggplot(data=time_ROC_df)+  geom_line(aes(x=FP_1year,y=TP_1year),size=1,color="#BC3C29FF")+  geom_line(aes(x=FP_3year,y=TP_3year),size=1,color="#0072B5FF")+  geom_line(aes(x=FP_5year,y=TP_5year),size=1,color="#E18727FF")+  geom_abline(slope=1,intercept=0,color="grey",size=1,linetype=2)+  theme_bw()+  theme(panel.grid=element_blank())+  annotate("text",x=0.75,y=0.25,size=4.5,           label=paste0("AUC at 1 year = 0.868 (0.840~0.896)"),color="#BC3C29FF")+  annotate("text",x=0.75,y=0.15,size=4.5,           label=paste0("AUC at 3 years = 0.862 (0.840~0.884)"),color="#0072B5FF")+  annotate("text",x=0.75,y=0.05,size=4.5,           label=paste0("AUC at 5 years = 0.829 (0.802~0.855)"),color="#E18727FF")+  labs(x="1-Specificity",y="Sensitivity")+  theme(axis.text=element_text(face="bold",size=11,color="black"),        axis.title.x=element_text(face="bold",size=14,color="black",margin=margin(c(15,0,0,0))),        axis.title.y=element_text(face="bold",size=14,color="black",margin=margin(c(0,15,0,0))))f1<-cph(Surv(months,os)~Age+Race+Chemotherapy+Year+Summary_stage+          Surg_Prim_Site,x=T,y=T,surv=T,data=rt,time.inc=12)pred_f_training<-predict(f1,rt,type="lp")data_table<-data.frame(time=rt[,"months"],status=rt[,"os"],score=pred_f_training)time_roc_res1<-timeROC(T=data_table$time[1:1000],                       delta=data_table$status[1:1000],                       marker=data_table$score[1:1000],                       cause=1,weighting="marginal",                       times=c(12,36,60),ROC=T,iid=T)compare(time_roc_res,time_roc_res1,adjusted=T)##fit<-survfit(Surv(months,CODKM1)~Race,data=rt)surv_diff<-survdiff(Surv(months,CODKM1)~Race,data=rt)ggsurvplot(fit,data=rt,fun='event',palette="set6",           cumevents=T,pval=T,pval.coord=c(60,0.4),ylim=c(0,1),           ylab="Cumulative hazard",xlab="Time(months)",           legend.labs=c("White","Black","Asian or Pacific Islander","American Indian/Alaska Native"),           legend.title="Race")fit<-survfit(Surv(months,os)~Race,data=rt)fit<-survfit(Surv(months,os)~Race,data=rt)surv_diff<-survdiff(Surv(months,os)~Race,data=rt)ggsurvplot(fit,data=rt,fun='event',palette="set6",           cumevents=T,pval=T,pval.coord=c(60,0.4),ylim=c(0,1),           ylab="Cumulative hazard",xlab="Time(months)",           legend.labs=c("White","Black","Asian or Pacific Islander","American Indian/Alaska Native"),           legend.title="Race")fit<-survfit(Surv(months,css)~Race,data=rt)fit<-survfit(Surv(months,css)~Race,data=rt)surv_diff<-survdiff(Surv(months,css)~Race,data=rt)ggsurvplot(fit,data=rt,fun='event',palette="set6",           cumevents=T,pval=T,pval.coord=c(60,0.4),ylim=c(0,1),           ylab="Cumulative hazard",xlab="Time(months)",           legend.labs=c("White","Black","Asian or Pacific Islander","American Indian/Alaska Native"),           legend.title="Race")
