# Supplementary material for: Cardiovascular mortality risk in patients with ovarian cancer: a population-based study
Source: J Ovarian Res. 2024 Apr 25;17:88. doi: 10.1186/s13048-024-01413-4 (PMC11044491; doi:10.1186/s13048-024-01413-4)
Supplement: Supplementary file 3 — Additional File 3. Table S1. Baseline characteristics of CVD in patients with ovarian cancer. Table S2. Competing risk regression analysis for predictors of cardiovascular mortality in patients with ovarian cancer. [file 13048_2024_1413_MOESM3_ESM.docx]

TableS1. Baseline characteristics of CVD in patients with ovarian cancer.

| **Characteristics** | **All (N=88653)** | **No CVD (N=86371** | **CVD (N=2282)** | ***p*-value** |
| --- | --- | --- | --- | --- |
| **Age** |  |  |  | <0.001 |
| 01-14years | 480 (0.54%) | 478 (0.55%) | 2 (0.09%) |  |
| 15-29years | 2454 (2.77%) | 2447 (2.83%) | 7 (0.31%) |  |
| 30-44years | 7792 (8.79%) | 7743 (8.96%) | 49 (2.15%) |  |
| 45-49years | 25822 (29.1%) | 25536 (29.6%) | 286 (12.5%) |  |
| 60-74years | 37250 (42.0%) | 36285 (42.0%) | 965 (42.3%) |  |
| 75+years | 14855 (16.8%) | 13882 (16.1%) | 973 (42.6%) |  |
| **Race** |  |  |  | <0.001 |
| American Indian/Alaska Native | 586 (0.66%) | 572 (0.66%) | 14 (0.61%) |  |
| Asian or Pacific Islander | 7059 (7.96%) | 6928 (8.02%) | 131 (5.74%) |  |
| Black | 7082 (7.99%) | 6865 (7.95%) | 217 (9.51%) |  |
| White | 73926 (83.4%) | 72006 (83.4%) | 1920 (84.1%) |  |
| **Chemotherapy** |  |  |  | <0.001 |
| No/Unknown | 28715 (32.4%) | 27559 (31.9%) | 1156 (50.7%) |  |
| Yes | 59938 (67.6%) | 58812 (68.1%) | 1126 (49.3%) |  |
| **Summary stage** |  |  |  | <0.001 |
| Distant | 52082 (58.7%) | 50938 (59.0%) | 1144 (50.1%) |  |
| Localized | 15295 (17.3%) | 14770 (17.1%) | 525 (23.0%) |  |
| Regional | 18641 (21.0%) | 18112 (21.0%) | 529 (23.2%) |  |
| Unknown/unstaged | 2635 (2.97%) | 2551 (2.95%) | 84 (3.68%) |  |
| **Surgery** |  |  |  | <0.001 |
| Cytoreductive surgery | 32263 (36.4%) | 31638 (36.6%) | 625 (27.4%) |  |
| No surgery | 11968 (13.5%) | 11588 (13.4%) | 380 (16.7%) |  |
| Other | 878 (0.99%) | 855 (0.99%) | 23 (1.01%) |  |
| Palliative surgery | 43544 (49.1%) | 42290 (49.0%) | 1254 (55.0%) |  |
| **ICD-O-3** |  |  |  | <0.001 |
| Adenocarcinoma | 75094 (84.7%) | 73179 (84.7%) | 1915 (83.9%) |  |
| Epithelial carcinoma | 3871 (4.37%) | 3772 (4.37%) | 99 (4.34%) |  |
| Other type | 6454 (7.28%) | 6317 (7.31%) | 137 (6.00%) |  |
| Sarcoma | 3234 (3.65%) | 3103 (3.59%) | 131 (5.74%) |  |
| **Year of diagnose** |  |  |  | <0.001 |
| 2000-2005 | 29248 (33.0%) | 28068 (32.5%) | 1180 (51.7%) |  |
| 2006-2011 | 29676 (33.5%) | 28936 (33.5%) | 740 (32.4%) |  |
| 2012-2017 | 29729 (33.5%) | 29367 (34.0%) | 362 (15.9%) |  |

TableS2. Competing risk regression analysis for predictors of cardiovascular mortality in patients with ovarian cancer.

| **Characteristics** | **Univariate analysis** | | **Multivariate analysis** | | | |  |
| --- | --- | --- | --- | --- | --- | --- | --- |
|  | **Adjusted HR (95%CI)** | ***p*-value** | **Adjusted HR (95%CI)** | | ***p*-value** | |  |
| **Age** |  |  |  | |  | |  |
| 01-14 years | Reference |  |  | |  | |  |
| 15-29 years | 0.22 (0.03-1.55) | 0.128 | 0.19 (0.03-1.32) | | 0.093 | |  |
| 30-44 years | 1.32 (0.32-5.48) | 0.706 | 0.98 (0.23-4.11) | | 0.976 | |  |
| 45-49 years | 2.74 (0.68-11.03) | 0.157 | 2.03 (0.50-8.26) | | 0.322 | |  |
| 60-74 years | 7.94 (1.98-31.79) | 0.003 | 5.67 (1.40-22.90) | | 0.015 | |  |
| 75+ years | 36.76 (9.17-147.38) | <0.001 | 21.07 (5.21-85.30) | | <0.001 | |  |
| **Race** |  |  |  |  | |  | |
| White | Reference |  |  |  | |  | |
| Black | 1.41 (1.22-1.62) | <0.001 |  | 1.50 (1.30-1.73) | | <0.001 | |
| Asian or Pacific Islander | 0.66 (0.55-0.79) | <0.001 |  | 0.88 (0.74-1.06) | | 0.176 | |
| American Indian/Alaska Native | 0.98 (0.58-1.66) | 0.944 |  | 1.34 (0.79-2.26) | | 0.281 | |
| **Chemotherapy** |  |  |  |  | |  | |
| No | Reference |  |  |  | |  | |
| Yes | 0.53 (0.49-0.57) | <0.001 |  | 0.48 (0.44-0.52) | | <0.001 | |
| **Summary stage** |  |  |  |  | |  | |
| Distant | Reference |  |  |  | |  | |
| Localized | 0.66 (0.60-0.74) | <0.001 |  | 0.66 (0.58-0.75) | | <0.001 | |
| Regional | 0.69 (0.62-0.76) | <0.001 |  | 0.78 (0.70-0.88) | | <0.001 | |
| Unknown/unstaged | 1.08 (0.86-1.35) | 0.512 |  | 0.63 (0.50-0.80) | | <0.001 | |
| **Surgery** |  |  |  |  | |  | |
| Cytoreductive surgery | Reference |  |  |  | |  | |
| No | 4.11 (3.61-4.68) | <0.001 |  | 2.77 (2.41-3.18) | | <0.001 | |
| Other | 1.21 (0.80-1.84) | 0.368 |  | 1.17 (0.76-1.79) | | 0.473 | |
| Palliative surgery | 0.89 (0.81-0.98) | 0.022 |  | 1.01 (0.90-1.12) | | 0.910 | |
| **ICD-O-3** |  |  |  |  | |  | |
| Adenocarcinoma | Reference |  |  |  | |  | |
| Epithelial carcinoma | 1.41 (1.15-1.72) | <0.001 |  | 0.98 (0.79-1.20) | | 0.815 | |
| Other types | 0.58 (0.49-0.69) | <0.001 |  | 0.70 (0.58-0.83) | | <0.001 | |
| Sarcoma | 2.35 (1.97-2.80) | <0.001 |  | 1.99 (1.67-2.38) | | <0.001 | |
| **Year of diagnose** |  |  |  |  | |  | |
| 2000-2005 | Reference |  |  |  | |  | |
| 2006-2011 | 0.86 (0.78-0.95) | 0.003 |  | 0.91 (0.82-1.00) | | 0.054 | |
| 2012-2017 | 0.75 (0.66-0.86) | <0.001 |  | 0.79 (0.69-0.90) | | <0.001 | |
